# Proteomic endorsed transcriptomic profiles of venom glands from *Tityus obscurus* and *T*. *serrulatus* scorpions

**DOI:** 10.1371/journal.pone.0193739

**Published:** 2018-03-21

**Authors:** Ursula Castro de Oliveira, Milton Yutaka Nishiyama, Maria Beatriz Viana dos Santos, Andria de Paula Santos-da-Silva, Hipócrates de Menezes Chalkidis, Andreia Souza-Imberg, Denise Maria Candido, Norma Yamanouye, Valquíria Abrão Coronado Dorce, Inácio de Loiola Meirelles Junqueira-de-Azevedo

**Affiliations:** 1 Laboratório Especial de Toxinologia Aplicada, CeTICS, Instituto Butantan, São Paulo, São Paulo, Brazil; 2 Laboratório de Farmacologia, Instituto Butantan, São Paulo, São Paulo, Brazil; 3 Faculdades Integradas do Tapajós, /Faculdade da Amazônia, Santarém, Pará, Brazil; 4 Laboratório de Artrópodes, Instituto Butantan, São Paulo, São Paulo, Brazil; Universidad de Costa Rica, COSTA RICA

## Abstract

**Background:**

Except for the northern region, where the Amazonian black scorpion, *T*. *obscurus*, represents the predominant and most medically relevant scorpion species, *Tityus serrulatus*, the Brazilian yellow scorpion, is widely distributed throughout Brazil, causing most envenoming and fatalities due to scorpion sting. In order to evaluate and compare the diversity of venom components of *Tityus obscurus* and *T*. *serrulatus*, we performed a transcriptomic investigation of the telsons (venom glands) corroborated by a shotgun proteomic analysis of the venom from the two species.

**Results:**

The putative venom components represented 11.4% and 16.7% of the total gene expression for *T*. *obscurus* and *T*. *serrulatus*, respectively. Transcriptome and proteome data revealed high abundance of metalloproteinases sequences followed by sodium and potassium channel toxins, making the toxin core of the venom. The phylogenetic analysis of metalloproteinases from *T*. *obscurus* and *T*. *serrulatus* suggested an intraspecific gene expansion, as we previously observed for *T*. *bahiensis*, indicating that this enzyme may be under evolutionary pressure for diversification. We also identified several putative venom components such as anionic peptides, antimicrobial peptides, bradykinin-potentiating peptide, cysteine rich protein, serine proteinases, cathepsins, angiotensin-converting enzyme, endothelin-converting enzyme and chymotrypsin like protein, proteinases inhibitors, phospholipases and hyaluronidases.

**Conclusion:**

The present work shows that the venom composition of these two allopatric species of *Tityus* are considerably similar in terms of the major classes of proteins produced and secreted, although their individual toxin sequences are considerably divergent. These differences at amino acid level may reflect in different epitopes for the same protein classes in each species, explaining the basis for the poor recognition of *T*. *obscurus* venom by the antiserum raised against other species.

## 1. Background

With more than 200 described species distributed in Central America and South America, genus Tityus (Koch, 1836), family Buthidae, contains the greatest number of species among the 13 extant scorpion families recognized to date [1]. Over 50,000 cases of scorpionism were registered in Brazil in 2015 with 77 deaths [2–5]. In Brazil, this genus is mainly represented by the medically important species *T*. *serrulatus*, *T*. *bahiensis*, *T*. *obscurus* and *T*. *stigmurus*. While *T*. *serrulatus* is widely distributed in Brazil, being responsible for most accidents by scorpions in the country and thus, intensively studied, *Tityus obscurus* is only found in the northern region, where it ranks as the second leading agent of accidents by venomous animals in the state of Pará, in the Amazon region [6–8]. *Tityus obscurus* (Gervais, 1843) is known as the Amazonian black scorpion and is synonym of *T*. *cambridgei* Pocock (1897) and *Tityus paraensis* Kraepelin, 1896 [7]. In general, clinical manifestations of *Tityus obscurus* sting include local pain, erythema, and effects on the autonomous nervous system such as hypertension, tachycardia, sweating and sialorrhea and it is particularly fatal for infant victims [9,10]. *T*. *obscurus* sting also causes neurological manifestations such as ataxia, dysmetria and symptoms described as “electrical shock”, which causes muscular contraction of the body [10,11]. Nevertheless, there are some differences between symptoms described in accidents with animals from different locations [9]. *Tityus serrulatus* is popularly known as the yellow scorpion and since it causes most accidents [5], the envenoming by this scorpion is the most studied [12,13]. Envenomation may present local pain, sweating, nauseas, tachycardia, tachypnea, hypertension, and in severe cases cardiac failure, lung edema, convulsions and coma [6].

Scorpion venom, in general, contains a variety of molecules, and its neurotoxins are the major compounds responsible for the symptoms of envenomation [14]. Some of the toxins, particularly those that modulate ion channel activity [6], are classified according to their affinity to ion channels. They may act as toxic depressants or excitatory molecules for arthropods, and they may also be toxic to mammals. Neurotoxins are involved in capturing prey and acting as defense against predators [15–17]. Other classes of venom components have different activities and functions such as antimicrobial peptides, bradykinin-potentiating peptide, hypotensins, anionic peptides, metalloproteinases, serine proteinases and proteinases inhibitors.

*T*. *serrulatus* venom has been extensively studied, mainly the sodium and potassium channel toxin [12,13,18,19]. Other components with low molecular mass such as hypotensins, antimicrobial peptides, bradykinin-potentiating peptides and high molecular mass such as enzymatic components like hyaluronidases, serino proteinases, metalloproteinase and proteinase inhibitors were also detected in *T*. *serrulatus* venom through biochemical isolation, transcriptomic and proteomic approaches [20–30].

Specifically regarding *T*. *obscurus* venom, there are few reports available on ion channel neurotoxins, and most studies have described potassium and sodium channel toxins through biochemistry and protein sequencing analysis. Batista and colleagues [31,32] have characterized the first potassium (Tc1) channel toxin using amino acid sequencing and mass spectrometry from *T*. *obscurus* venom. Later, a proteomic study of the soluble part of *T*. *obscurus* venom was performed after the separation of 60 different compounds by high-performance liquid chromatography (HPLC); 26 components had the N-terminal sequenced by Edman´s degradation and 5 were entirely sequenced [33]. This study did not elucidate all the venom components separated by HPLC; they focused on the ion channel peptides that are the toxic fraction responsible for the most important envenomation symptoms and affect the excitable and non-excitable cells.

The arsenals of toxins present in scorpion venoms have been described mostly for toxins obtained from the transcriptomes of venom glands or from venom proteomes [30,34–44]. In recent years, there is a growing tendency to combine transcriptome and proteome studies for characterizing scorpion venoms [45–53], but rarely are both approaches used at the same time for *Tityus* scorpions. A recurrent problem of transcriptome-only based characterizations of scorpion venom glands is the inherent uncertainty in distinguishing transcript coding real venom proteins from those coding endophysiological proteins acting inside the venom gland or in the surrounding tissues of the telson or secreted to the hemolymph [54]. On the other hand, proteomic characterizations by spectrometric analysis are quite dependent on species-specific sequence databases for matching MS spectral profiles. If unspecific datasets are used, the identification profile may be biased towards common or conserved components, which could be particularly problematic for species with long-time divergence, such as scorpions.

The present work shows the transcriptomic profiles of the venom glands from the scorpions *Tityus obscurus* and *T*. *serrulatus* based on high-throughput sequencing of its cDNAs, corroborated by the proteomic identification of the proteins and peptides secreted into the venom from *T*. *obscurus* and *T*. *serrulatus*.

## 2. Results and discussion

### 2.1 Transcriptomic profile of venom gland components

**Fig 1:**
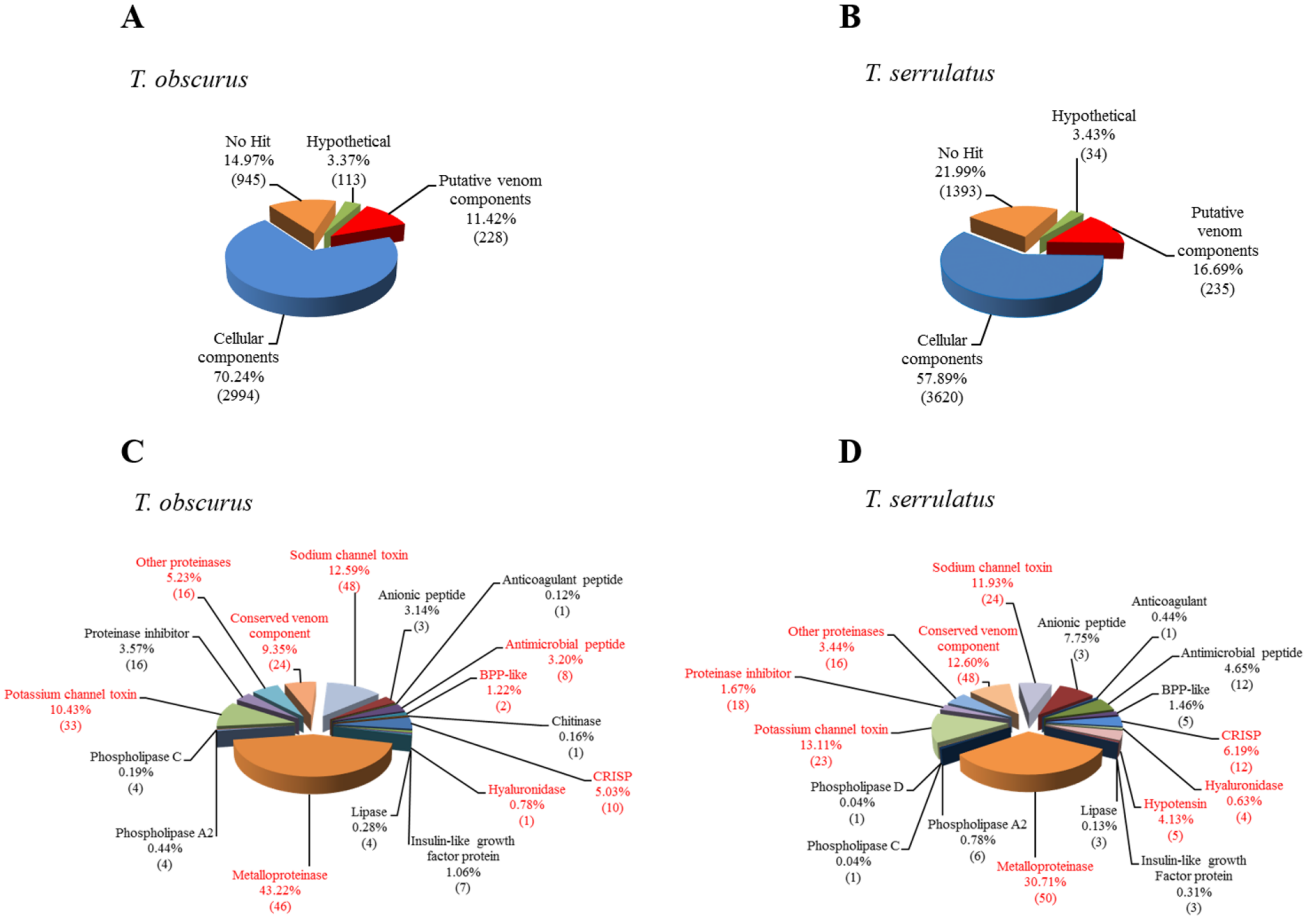
Transcripts from the venom gland of *Tityus obscurus and Tityus serrulatus*. Functional classification of the transcripts (A) *T*. *obscurus* and (B) *T*. *serrulatus*. Relative proportion of each category of venom components. The value between parentheses indicates the number of isotigs in each category. Categories in red are those with peptides identified by proteomic analysis (C) *T*. *obscurus* and (D) *T*. *serrulatus*.

Sequencing the venom gland transcriptome of *T*. *obscurus and T*. *serrulatus* resulted in 102,428 and 165,646 high-quality filtered reads. Assembling using Newbler software produced 4,280 and 5,282 isotigs that represent putative transcripts (S1 Table). We performed an automatic search using a BlastX alignment tool and an annotation using Blast2GO [55], in order to assign putative venom components, cellular components, hypothetical proteins and non identified sequences (Fig 1A and 1B). The global expression profile of the sample was calculated using the CLC Genomics Workbench by counting the reads mapped back to the isotigs and normalizing the count according to the RPKM (reads per kilobase per million reads mapped) conversion [56] in order to remove biases inherent in the sequencing approaches, such as the length of the RNA species and the sequencing depth of sample.

*T*. *obscurus* had 70.24% and *T*. *serrulatus* 57.89% of transcripts coded for cellular components, whereas hypothetical proteins/peptides accounted for 3.37% (*T*. *obscurus*) and 3.43% (*T*. *serrulatus*). In *T*. *obscurus* and *T*. *serrulatus* some sequences (14.97% and 21.99%, respectively) had no hit in Blast searches, possibly representing specific transcripts of this species (Fig 1A and 1B). The putative venom components from *T*. *obscurus* represented 11.42% and *T*. *serrulatus* 16.69% of the expression level of the transcripts, which could be grouped into 17 and 18 categories of encoded proteins, respectively (Fig 1C and 1D). The expression levels of transcripts coding for putative venom components were lower than other scorpion transcriptome analysis [34,42,48], though very similar to *T*. *bahiensis* transcriptome sequenced using the same approach [43].

We checked if the high expression of transcripts coding for cellular proteins could be related to the fact that those transcripts are larger, on average, than toxin transcripts, and thus biased by the higher production of sequencing reads from longer RNAs during NGS library preparation. We noticed, however, that the RPKM normalization efficiently applied corrected this bias by improving the expression values of shorter transcripts (blue bars in S1 and S2 Figs) and reducing them in larger transcripts. Accordingly, among the largest transcripts, many are highly expressed and many have low expression. In fact, some transcripts coding for muscle specific and metabolism marker proteins such as cytochrome oxidase, myosin and actin were very abundant, thus indicating that muscle cell transcripts influence the transcriptional profile of the telson. Nevertheless, we previously noticed the possibility that the 454 library prep protocol causes some loss of very small transcripts, such as those of short neurotoxins, thus contributing to a possible underestimation of venom components [43].

The use of telsons removed 48 hours after venom extraction is the standard for transcriptomic analysis [36,45,57] as there is evidence that this is the peak of mRNA production [58,59]. In the transcriptome of the venom gland from *Centruroides noxius*, also performed by 454, the authors reported that the resting gland expression profile was lower in contrast to the replenishing gland [60]. Luna-Ramírez and colleagues (2015) [44] recently analyzed the transcriptomic profile of *Urodacus yaschenkoi* scorpion using an Illumina platform and described 210 transcripts coding for 111 unique venom compounds, which is in agreement with the 228 and 235 transcripts found for *T*. *obscurus and T*. *serrulatus*, respectively (S2 and S3 Tables). The Transcriptome Shotgun Assembly projects were deposited at the DDBJ/EMBL/GenBank under the accession GEMQ00000000 (*T*. *obscurus*) and GEUW00000000 (*T*. *serrulatus*). The versions described in this paper are the first version, GEMQ01000000 (*T*. *obscurus*), and GEUW01000000 (*T*. *serrulatus*).

### 2.2 The proteomic identification of *Tityus* venom components

Our proteomic analysis for the two *Tityus* species identified peptides that mapped onto isotigs coding for miscellaneous putative venom components (S4 and S5 Tables), such as sodium and potassium channel toxins, metalloproteinases, hyaluronidase, cysteine-rich protein, and trypsin-like protein. The proteomic analysis also detected other protein classes unexpected in venom such as angiotensin-converting enzyme and endothelin-converting enzyme, whose roles in the venom are unclear (Fig 1C and 1D- conserved venom components). It is important to note that we considered a protein identification even if only one peptide-spectrum match was obtained, since part of the proteins expected in the venom (such as ion channel toxins) are small. However, those cases are marked in red in Supplementary S4 and S5 Tables and the images of individual spectra are provided in the tables.

Mass-spectrometry-based proteomics has allowed the identification of new venom components of several scorpions [29,46,49,54,61–68], especially ion channel modulating peptides from *T*. *serrulatus* with amino acids sequenced. Enzymes with gelatinolytic activity [24], metalloproteinase [25,26] and proteinase inhibitors [27] have also been described and had their amino acid sequence resolved. In a mass fingerprint approach of toxic fractions (low molecular masses) from venom of *T*. *serrulatus*, Pimenta et al. (2001) [29] detected sodium and potassium channel toxins and unknown peptides (molecular masses ranging from 2500 to 7500 Da). In 2008, Rates and colleagues [40] accessed *T*. *serrulatus* venom peptidomics, identifying around 28 peptides as fragments from Pape proteins, scorpion-like, potassium channel toxins, hypotensins and novel peptides with no identification in the Swiss-Prot database.

Other venom components of *T*. *serrulatus* scorpions had their amino acid sequences elucidated as antimicrobial peptides [40], hypotensins [20], C-type natriuretic peptide [69], hyaluronidase [70], metalloproteinase [25,26], non-disulfide-bridged peptides with angiotensin-converting enzyme inhibitor activity [27], neprilysin-like enzyme inhibitor [71] and angiotensin-converting enzyme-like peptidase [72]. Batista and colleagues (1998) [73] first described *T*. *obscurus* venom components and during the following years described 3 potassium channel and 22 sodium channel toxins based on mass spectrometry approach and physiological analysis [31–33,74,75]. In 2012, Guerrero-Vargas [17] and colleagues described 15 more sequences of sodium channel toxins based on cDNA sequencing and mass spectrometry analysis.

We also detected some components in our proteomic analysis that we identified as contaminants of the venom gland, such as carbonic anhydrase, alpha-2-macroglobulin, myostatin, peptidylglycine alpha-amidating monooxygenase, nucleoredoxin-like and transferrin. These putative components have lower expression levels, with the exception of transferrin, which had high expression level in the transcriptomic analysis. Peptides that mapped hemocyanins, carcinolectin and tachylectin were also detected in proteomics. However, these components are known “contaminants” that are present in the venom mixture [54,76] and were not represented in Table 1 or in Fig 1C and 1D. Table 1 summarizes the putative venom components detected based on transcriptomic and proteomic evidence for *T*. *obscurus* and *T*. *serrulatus*.

**Table 1:** Putative venom components detected in this work based on transcriptomic and proteomic evidence for *T*. *obscurus* and *T*. *serrulatus*.

Number of transcripts *T*. *obscurus/T*. *serrulatus*	Number of transcripts with proteomic evidence *T*. *obscurus/T*. *serrulatus*	Putative activity	Putative conserved domain detected	Known/New *T*. *obscurus*	Known/New *T*. *serrulatus*
2/1	1/0	Angiotensin converting enzyme like	GluZincin super family	0/2	0/1
3/3	-	Anionic peptide	-	0/3	0/3
1/1	-	Anticoagulant peptide	Trypsin Inhibitor-like cysteine-rich domain	0/1	0/1
8/12	-	Antimicrobial peptide	-	0/8	0/12
2/5	-	BPP-like peptide	-	0/2	0/5
1/2	-	Chitinase	GH18_chitinase-like super family	0/1	0/2
1/4	-	Ctenitoxin-like	Thyroglobulin type I repeats	0/1	0/4
10/12	3/3	Cysteine-rich protein, allergen V5/Tpx-1-related	SCP-like extracellular protein domain	0/10	0/12
1/8	1/0	Endothelin-converting enzyme-like	Peptidase family M13 includes neprilysin	0/1	0/8
1/4	1/3	Hyaluronidase	Glyco_hydro_56, hyaluronidase	0/1	0/4
0/5	0/2	Hypotensin	-	0/0	3/2
7/3	0/1	Insulin-like growth factor-binding protein	Insulin growth factor-binding protein homologues	0/7	0/3
46/50	27/22	Metalloproteinase	Zinc-dependent metalloprotease, M12, the astacin-like proteases and the adamalysin/reprolysin-like proteases	0/46	7/43
16/16	2/1	Other proteinases (serine and cysteine proteinases)	Trypsin-like serine protease, Clip or disulphide knot domain, Cathepsin B group; Cathepsin_D_like; Cysteine protease, C1A family	0/16	0/16
4/3	-	Pancreatic lipase-like protein	Pancreatic lipase-like enzymes.	0/4	0/3
4/6	1/0	Phospholipase A2	Phospholipase A2	0/4	0/6
4/1	-	Phospholipase C	Pleckstrin homology-like domain and EF-hand, calcium binding motif	0/4	0/1
0/1	-	Phospholipase D	Phospholipase D	0/0	0/1
33/23	1/6	Potassium channel toxin	This family includes scorpion potassium channel toxins with 4 conserved cysteine	1/32	6/17
15/18	1/1	Proteinase inhibitors	Serine Proteinase Inhibitors (serpins), or Kunitz, Kazal type or metalloproteinase inhibitors	0/15	0/18
48/24	3/7	Sodium channel toxin	Scorpion toxin-like domain. This family contains both neurotoxins and plant defensins.	9/39	9/15

### 2.3 The ion channel toxins

**Fig 2:**
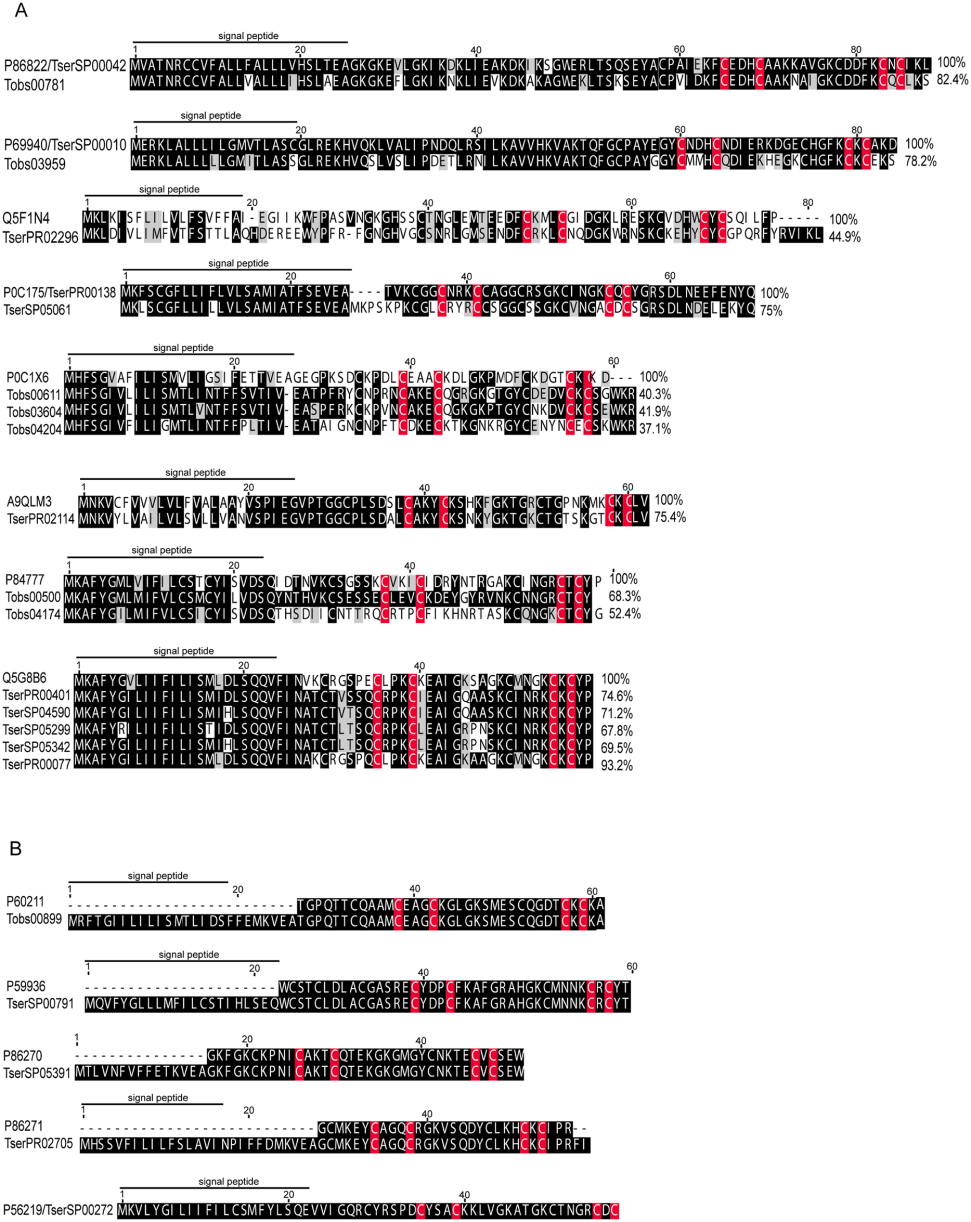
Alignment of amino acid sequences of putative potassium channel toxins from *T*. *obscurus* (Tobs) and *T*. *serrulatus* (Tser) with known toxins from *Tityus* scorpions. Variations in gray scale indicate levels of sequence conservation. The percentages of identity compared to the top sequence are indicated at the end of the alignment. The symbol (-) represents gaps to improve the alignment. The CxxxC and CxC motifs are indicated in red and the putative signal peptide is underlined. (A) shows the alignment of identical and similar sequences from *T*. *obscurus* and T. *serrulatus*, (B) shows identical and putative precursor sequences of *T*. *obscurus* and T. *serrulatus*. P86822—Ktx 2 from *T*. *serrulatus*, P69940—TsTXK-beta from *T*. *serrulatus*, Q5F1N4—potassium channel toxin BmTxKS4 from *Mesobuthus martensii*, P0C175—TsPep2 *T*. *serrulatus*, P0C1X6—potassium channel toxin from *T*. *discrepans*, A9QLM3—KTx8 from *Lychas mucronatus*, P84777—potassium channel toxin from *T*. *discrepans*, Q5G8B6—alpha-KTx 4.5 from *Tityus costatus*, P60211—potassium channel toxin alpha-KTx 18.1 from *T*. *obscurus*, P59936, P86270, P86271 are potassium channel toxins KTx 12.1, KTx 21.1 and Ts16 from *T*. *serrulatus*, respectively.

Potassium channel acting peptides are one of the most studied types of scorpion toxins and they are particularly well known in this species. Here we report on 33 and 23 isotigs with similarities with known potassium channel toxins from *T*. *obscurus* and *T*. *serrulatus*, respectively, and they represent 10.43% and 13.11% of the putative toxins. There are 13 different groups of putative potassium channel toxins in these transcriptomes. The first group showed similarities with the potassium channel toxin from *T*. *serrulatus* (P86822); the second group is similar to potassium channel toxin from *T*. *serrulatus* (P69940); the third group containing one *T*. *serrulatus* isotig was similar to the potassium channel toxin BmTxKS4 from *Mesobuthus martensii* (Q5F1N4); the fourth group showed similarities with two isotigs of *T*. *serrulatus* that were similar to *T*. *serrulatus* TsPep2 (P0C175); the fifth group of *T*. *obscurus* isotigs showed similarities to a potassium channel toxin from *T*. *discrepans* (P0C1X6); the sixth group was a *T*. *serrulatus* isotig similar to KTx8 from *Lychas mucronatus* (A9QLM3); the seventh group contains the potassium channel toxin from *T*. *discrepans* (P84777); the eighth group is composed of five isotigs from *T*. *serrulatus*, similar to alpha-KTx 4.5 from *Tityus costatus* (Q5G8B6); the ninth group involves one isotig that probably is the precursor of the potassium channel toxin alpha-KTx 18.1 (P60211) described from *T*. *obscurus*; the tenth, eleventh and twelfth are probably the precursors of the potassium channel toxins KTx 12.1, KTx 21.1 and Ts16 from *T*. *serrulatus* (P59936, P86270, P86271); and we also detected an identical isotig of *T*. *serrulatus* with KTx 4.2 (P56219). Until now only three potassium channel toxins have been described for *T*. *obscurus*: Tc1 [31], Tc30 and Tc32 [74]. Fig 2 provides an alignment of an unique isotig with a high coverage sequence representing these groups. The proteomic analysis confirmed a predicted peptides for 7 isotigs (1 and 6 from *T*. *obscurus* and *T*. *serrulatus*, respectively), and the sequences of the peptides are indicated in S4 and S5 Tables.

We sequenced 48 and 24 transcripts (12.59% and 11.93% of putative toxins) that have similarities with sodium channel toxins from *T*. *obscurus* and *T*. *serrulatus*, respectively, including the sequences deposited in Genbank for both species. *T*. *obscurus* venom has lower toxicity (LD_50_ = 3.13 mg/kg) than *T*. *serrulatus* (LD_50_ = 0.99 mg/kg), but it can induce lethal activity [77]. The symptoms and behavioral effects in mice and rats were more intense at higher doses, but the envenoming in mice was less severe and non-convulsive compared to *T*. *serrulatus* [77]. The lower similarity of *T*. *obscurus* amino acid sequences with known toxins could explain differences in effects than those promoted by *T*. *serrulatus* venom, besides the symptoms described as “electrical shock” that occur only with *T*. *obscurus* venom. These data suggests that *T*. *obscurus* toxins could act in a specific ion channel. Therefore, this venom could be a good source for screening potential specific ion channel modulators.

**Fig 3:**
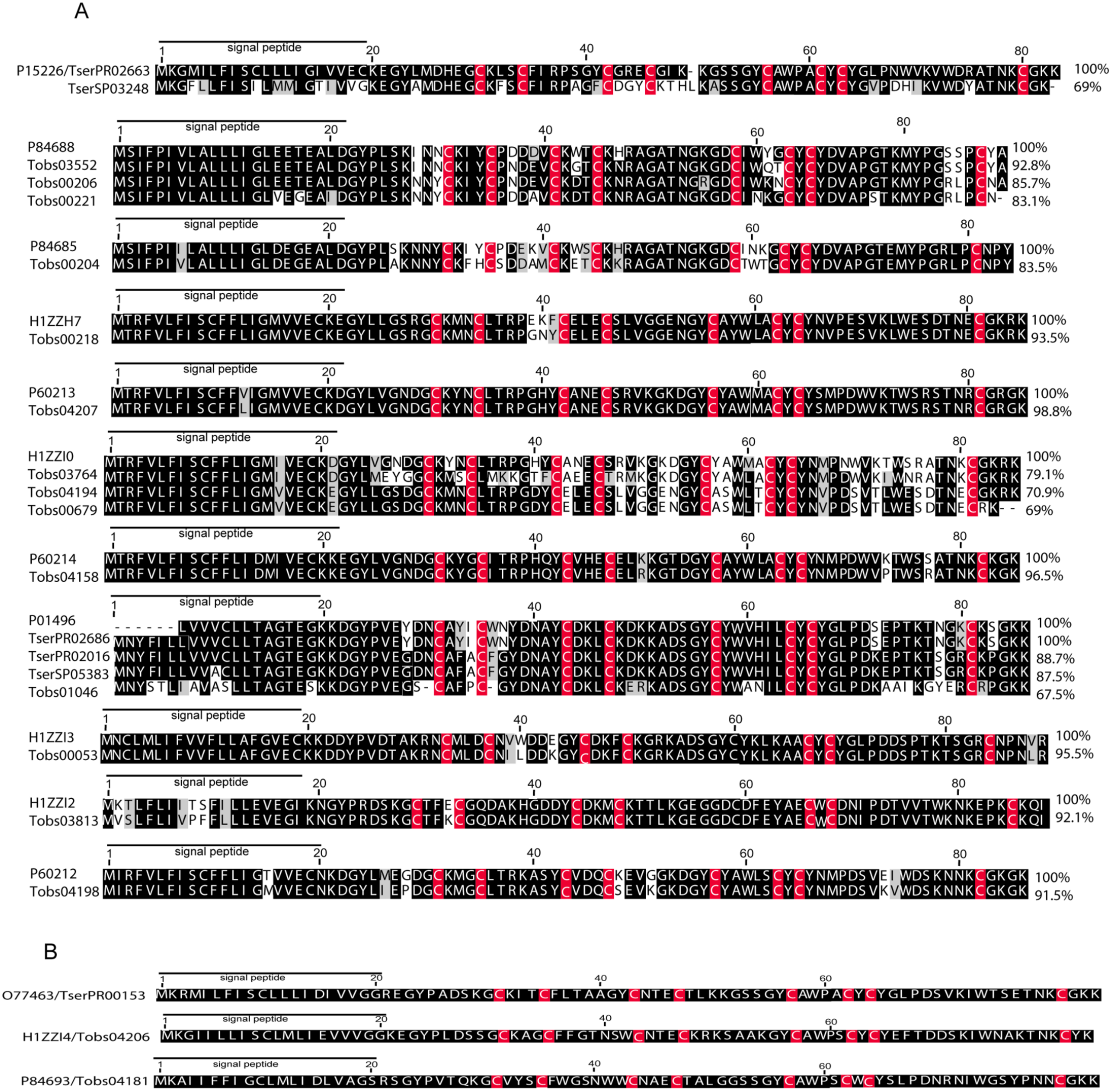
Alignment of the amino acid sequences of putative sodium channel toxins from *T*. *obscurus* (Tobs) and *T*. *serrulatus* (Tser) with known toxins from *Tityus* scorpions. Variations in gray scale indicate levels of sequence conservation. The percentages of identity compared to the top sequence are indicated at the end of the alignment. The symbol (-) represents gaps to improve the alignment. The putative signal peptide is underlined; the conserved cysteine residues are indicated in red; (A) shows the alignment of identical and similar sequences from *T*. *obscurus* and T. *serrulatus*, (B) shows identical sequences of *T*. *obscurus* and T. *serrulatus*. P15226—insect toxin Ts1 from *T*. *serrulatus*; P84688—Toxin To7 from *T*. *obscurus*; P84685—Toxin To6 from *T*. *obscurus*; H1ZZH7—Toxin To8 from *T*. *obscurus*; P60213—Toxin To3 from *T*. *obscurus*; H1ZZI0—Toxin To11 from *T*. *obscurus*; P60214—Toxin To1 from *T*. *obscurus*; P01496—Toxin-5 from *T*. *serrulatus*; H1ZZI3—Toxin To14 *T*. *obscurus*; H1ZZI2—Toxin To13 *T*. *obscurus*; P60212—toxin To2 from *T*. *obscurus*; O77463—Ts4 from *T*. *serrulatus*; H1ZZI4—Toxin To15 from *T*. *obscurus*; P84693—Toxin To5 from *T*. *obscurus*.

Our transcriptomic analysis revealed different types of sequences, those that were identical to previously described *T*. *obscurus* toxins To5 and To13 (Tobs 04181 and Tobs04206) and other transcripts showing distinct levels of similarities with the described *T*. *obscurus* toxins (groups second to sixth and eighth to eleventh of Fig 3). For *T*. *serrulatus*, our transcriptomic analysis showed sequences that were identical to previously described *T*. *serrulatus* (TserPR02663 and TserPR00153). The first group in Fig 3 has one isotig that is 69% identical to a Ts1—insect toxin from *T*. *serrulatus* (P15226). In the eighth group, we show three sequences from *T*. *serrulatus* (TserPR02016 and TserSP05583) and the isotig TserPR02686 is probably the precursor of Toxin-5. We also detected one sequence from *T*. *obscurus* (Tobs01046) with similarities to Toxin-5 from (P01496). Fig 3 provides an alignment of a unique isotig with a high coverage sequence representing these groups; the identity of each sequence with the known sequence reference is indicated. The proteomic analysis detected peptides that mapped to 3 isotigs from *T*. *obscurus*. For *T*. *serrulatus*, we detected peptides that mapped to 7 isotigs; the sequences of the peptides are shown in S4 and S5 Tables.

### 2.4 Metalloproteinases

Among the enzymatic components related to putative toxins, the metalloproteinases represented 43.22% and 30.71% of the total putative venom components of *T*. *obscurus* and *T*. *serrulatus*, respectively (Fig 1C and 1D). Likewise as we previously described for *T*. *bahiensis* [43], the metalloproteinases were the most abundant component identified in *T*. *obscurus* and *T*. *serrulatus* transcriptome. These results were also observed for venom glands transcriptomes from *T*. *serrulatus* [30] and for *Hottentotta judaicus* [37]. However, the relationship between these high levels of metalloproteinase expression has not yet been demonstrated in transcriptomes and the levels of proteins present in the venom. The proteomic analysis revealed a high number of peptides mapping to putative metalloproteinase transcripts.

Metalloproteinases have been identified in many animal venoms, being proteolytic enzymes whose activity is dependent on divalent ions, commonly a Zn^2+^ at the catalytic center. These enzymes may disrupt the cell matrix and the process of clotting blood or hemolymph. *T*. *obscurus* venom can cause lung damage characterized by the presence of red blood cells in the parenchyma [77]. The metalloproteinases found in this venom could contribute to these effects. The metalloproteinases from snake venom are multidomain enzymes known to be involved in inhibition of platelet aggregation, inflammation, apoptosis and hemorrhage [78]. In arthropods, metalloproteinases have been reported for many animal classes. *Tityus serrulatus* venom was described as proteolytic and its metalloproteinases were shown to be involved in pancreatic disturbances [25,79,80]. The metalloproteinases found here were shown to be Zn^2+^-dependent and related to vertebrate ADAM enzymes, a subtype of metzincin proteinases [26,43]. Carmo and colleagues (2014) [26] characterized metalloproteinases from *T*. *serrulatus* presenting activity in a fibrinogenic assay. Mature sequences of antarease-like enzymes were reported for other *Tityus* scorpions by Ortiz and co-workers (2014) [81] and we recently described several metalloproteinases from *T*. *bahiensis* [43]. The *T*. *obscurus* and *T*. *serrulatus* sequences, like other scorpion metalloproteinases, are shorter than typical ADAM enzymes and lack other domains such as the disintegrin present in snake venom metalloproteinases and cysteine-rich domains present in snake and acari metalloproteinases. Consequently, there is a possibility that this kind of metalloproteinase from scorpions might have evolved from an Arachnida type of ADAM-like ancestor [26,43] by losing the extra domains (disintegrin and cysteine-rich) in a similar trend towards simplification that is believed to have occurred with the PI-type metalloproteinases from snake venoms [82].

**Fig 4:**
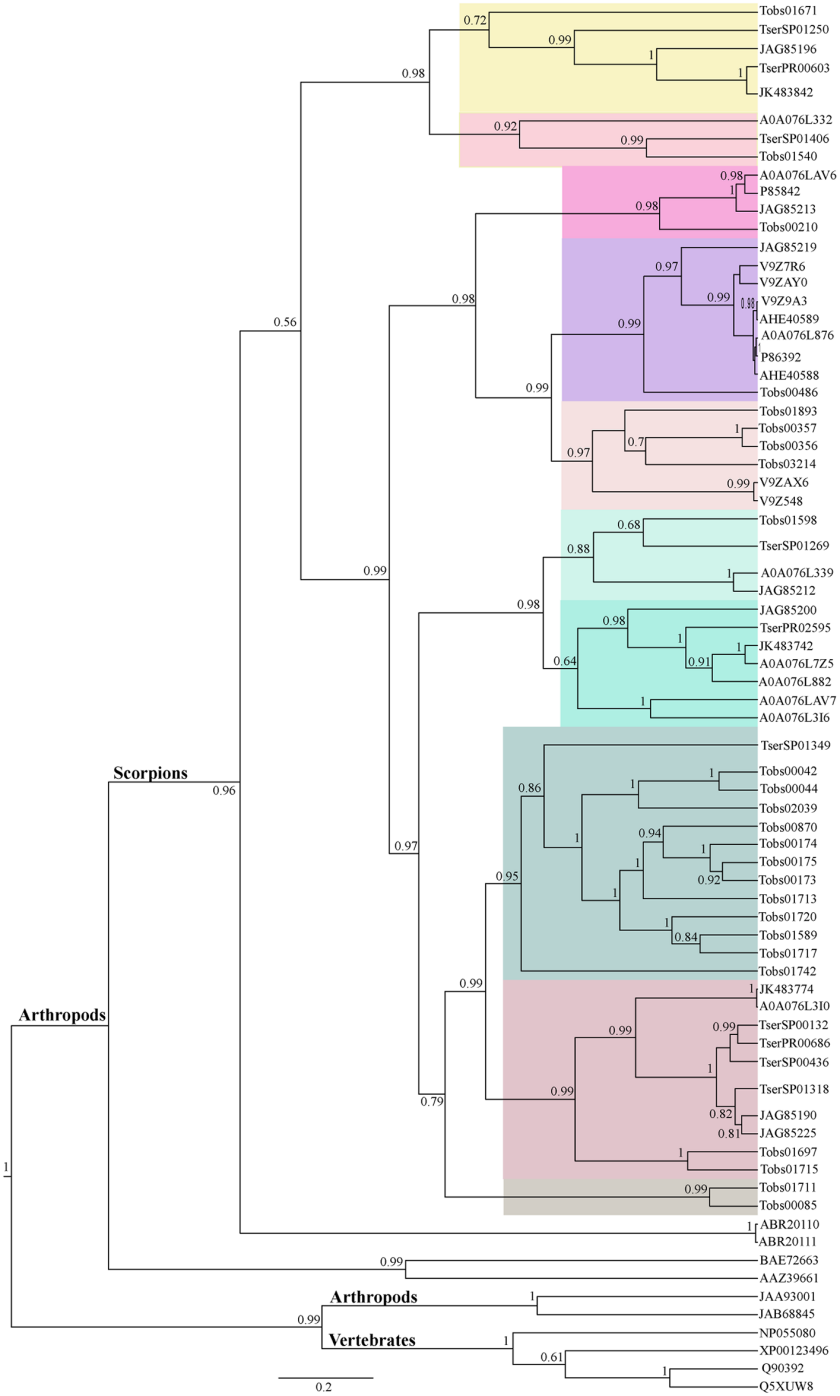
Bayesian phylogenetic analysis of putative metalloproteinases. The sequences from *T*. *obscurus* (Tobs) and *T*. *serrulatus* (Tser) obtained in this study and sequences from other scorpions, Arthropods (arachnida) and vertebrates are indicated and referred to their GenBank accession numbers. The colors in the cladogram represent 10 putative groups of paralogues from metalloproteinases shared between the *Tityus* genus. Vertebrates, Arthopods and scorpions are indicated. NP055080—ADAM 28 isoform 1 *Homo sapiens*, XP001233496—ADAM 28 isoform X1 *Gallus gallus*, Q5XUW8—Snake venom metalloproteinase insularinase-A, Q90392—Snake venom metalloproteinase atrolysin-C *Crotalus atrox*, BAE72663—metalloproteinase partial from *Haemaphysalis longicornis*, AAZ39661—salivary gland metalloproteinase *Rhipicephalus microplus*, JAA93001—putative ADAMTS *Cupiennius salei*, JAB68845—putative ADAMTS 7 *Ixodes ricinus*, ABR20110—venom metalloprotease-1 *Mesobuthus eupeus*, ABR20111—venom metalloprotease-2 *Mesobuthus eupeus*, P86392—venom metalloproteinase antarease from *T*. *serrulatus*, P85842—venom metalloproteinases from *T*. *serrulatus*, A0A076L876, A0A076LAV6, A0A076LAV7, A0A076L316, A0A076L339, A0A076L882, A0A076L7Z5, A0A076L3I0 and A0A076L332—*metalloserrulases from T*. s*errulatus*, V9Z9A3- venom metalloproteinase antarease-like from *T*. *serrulatus*, V9Z548 and V9ZAX6 –Venom metalloproteinase antarease-like from *T*. *pachyurus*, V9ZAY0—Venom metalloproteinase antarease-like from *T*. *trivittatus*, V9Z7R6—Venom metalloproteinase antarease-like from *T*. *fasciolatus*, JK483842, JK483742, JK483774—are *Tityus stigmurus*, similar to antarease, AHE40588 and AHE40589 are *T*. *serrulatus* antarease-like, JAG85190, JAG85190 and JAG85200 are putative venom metalloproteinase from *T*. *bahiensis*.

The phylogenetic reconstruction of *Tityus* metalloproteinases (Fig 4) showed that this group of scorpion metalloproteinases has the same phylogenetic origin and probably come from a gene duplication event. Scorpion metalloproteinases might be a sister clade of known metalloproteinases from Acari. Inside the scorpion clade, there are two major groups of metalloproteinases: one is more closely related and represents the majority of *Tityus* metalloproteinases (*T*. *bahiensis*, *T*. *fasciolatus*, *T*. *obscurus*, *T*. *pachyurus T*. *serrulatus*, *and T*. *trivittatus*) and the other contains sequences from *Mesobuthus*. The *T*. *obscurus* (Tobs) and *T*. *serrulatus* (Tser) sequences are distributed together with other *Tityus* species showing a high diversity of this group. The presence of at least ten putative paralogues can be observed in the phylogeny, represented by the grouping of orthologues from different species of the genus and one clade containing only two *T*. *obscurus* sequences. Thus, the diversity of metalloproteinase genes probably might have occurred during the speciation process, since some types of sequences are distributed in the *Tityus* genus and others are restricted to one species (Fig 4). The alignment used to generate the tree is presented in S3 Fig. The proteomic analysis revealed peptides that mapped to 27 and 22 isotigs of metalloproteinase transcripts from *T*. *obscurus* and *T*. *serrulatus*, respectively (S4 and S5 Tables).

### 2.5 Other venom components

Besides the major venom components, the transcriptomic profile of *T*. *obscurus* lead to the identification of antimicrobial peptides, anionic peptides, anticoagulant proteins, bradykinin-potentiating peptide, cysteine-rich secretory peptides, phospholipases A2 and C, lipases, proteinase inhibitors, serine and cysteine proteases, metalloproteinases and hyaluronidase. The proteome profile detected peptides matching isotigs coding for proteinases and proteinases inhibitors, hypotensins, hyaluronidases, CRISPs, ACE and ECE-like, serine proteinases and cathepsin-like.

We identified two angiotensin-converting enzyme-like molecules (ACE-like) in *T*. *obscurus* transcriptome (Tobs00978 and Tobs01141) and one in the *T*. *serrulatus* (TserSP00939). In scorpion transcriptomes, an ACE-like molecule was first described by Morgenstern and colleagues (2011) [37] from *Hottentotta judaicus* venom gland. Recently, Cajado-Carvalho and coworkers (2016) [72] isolated and characterized an ACE-like sequence from *T*. *serrulatus* venom that showed high similarities with *T*. *serrulatus and T*. *obscurus* ACE-like sequence. However, the ACE-like expression level is much lower than that of antarease-like metalloproteinases and in the proteomic results we identified 1 peptide that mapped to ACE-like isotig Tobs01141 (S4 Table).

The remaining conserved venom component is composed of those isotigs with high similarity with putative proteins sequenced from venom glands of other scorpions but not well characterized. Table 1 presents the list of venom components detected in the transcriptome and proteome of *T*. *obscurus* and *T*. *serrulatus*, including those not related to toxic functions.

### 2.6 *Tityus obscurus* venom components are not recognized by anti-*Tityus serrulatus* venom serum

**Fig 5:**
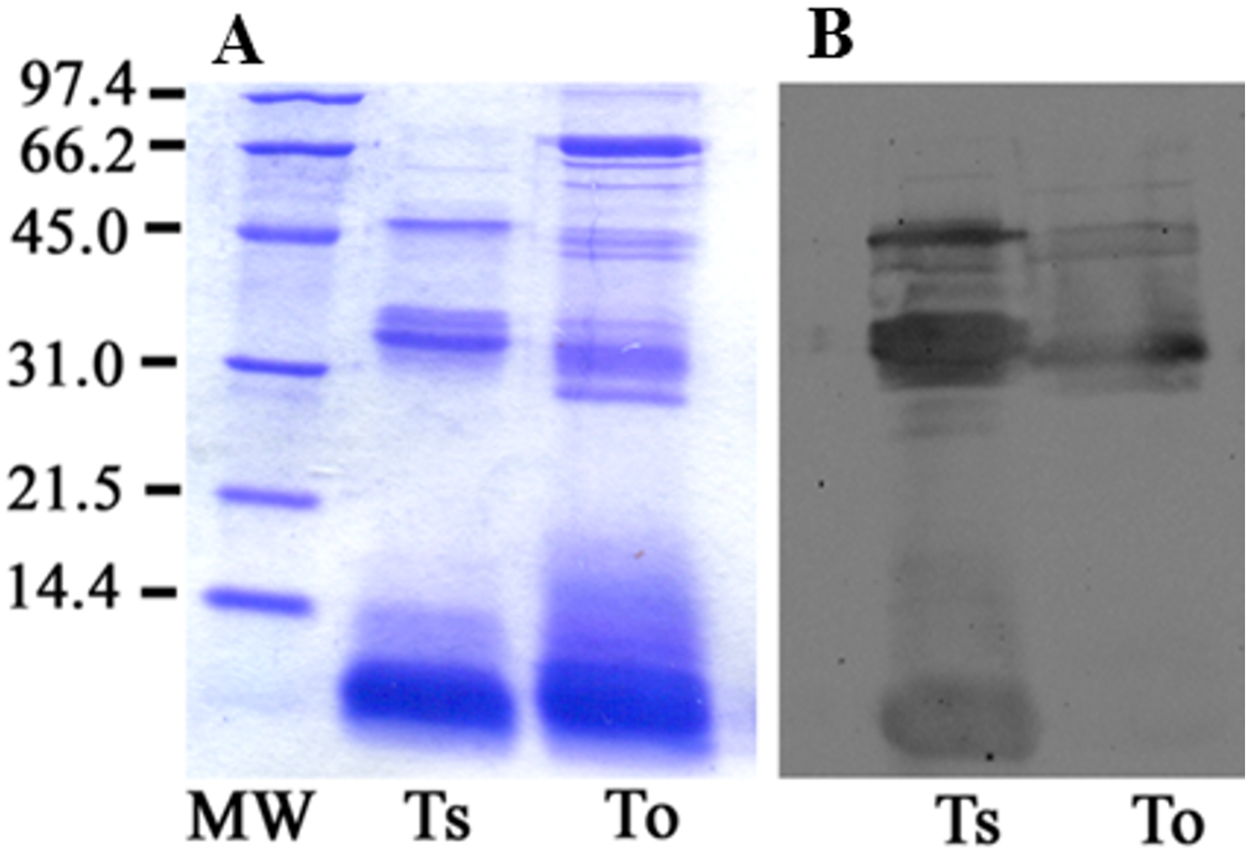
Differences between *Tityus serrulatus* (Ts) and *Tityus obscurus* (To) venom. (A) 1D SDS-PAGE of 30μg of each venom, stained with Coomassie Brilliant Blue R. (B) Western blotting using horse anti-*Tityus serrulatus* venom serum. Immunostained western blotting showing that anti-*Tityus serrulatus* venom serum did not recognize all toxins from *Tityus obscurus* (To) venom. Ts venom was used to compare the two venoms and as a positive control in Western blotting assay.

The eletrophoretic profile of *T*. *obscurus* venom revealed a major band below 14.4 kDa and the other components between 31.0 and 66.2 kDa, the *T*. *serrulatus* venom components had quite a different profile. Many components are located below 14.4 kDa in both venoms but significant differences were shown between these two venoms (Fig 5A).

The effectiveness of Brazilian anti-scorpionic serum (anti-*T*. *serrulatus*, produced by the Butantan Institute) has been demonstrated in neutralizing the most common *Tityus* species, such as *T*. *serrulatus*, *T*. *bahiensis*, *T*. *stigmurus* and *T*. *costatus* [83]. However, Amaral and Rezende (2000) [84] demonstrated that clinical symptoms and venom composition can change in different geographical regions for some *Tityus* species from South America, consequently affecting the efficacy of the antivenom.

The western blotting analysis of these venoms using a horse anti-*Tityus serrulatus* serum (Fig 5B) showed that *T*. *obscurus* venom components are not antigenically similar to *T*. *serrulatus*. These results are in consistent with the differences in clinical symptoms of these venoms [84,85], and with the transcriptome and proteome results described in this paper. As discussed for *Tityus* species from Venezuela [86], differences in cross reactivity of anti-*Tityus serrulatus* serum supports the regional sets of venom components, and the antigenic epitopes diverge considerably between unrelated *Tityus* species. It could result from the geographic location of this species, Amazon region, allowing the divergence of toxin repertories with distinct antigenic epitopes.

## 3. Conclusion

In conclusion, this work reports the first high-throughput sequencing of transcripts from venom glands of the Amazonian scorpion *Tityus obscurus*, corroborated by a proteomic identification of the proteins from their venom and by a comparison with *T*. *serrulatus* transcriptome and proteome. The omics analysis of both species led to the identification of not only new versions of the expected ion channel toxins but also different sorts of components such as metalloproteinases, IGFP-like, proteinases (serine and cysteine) and proteases inhibitors. A myriad of low abundance proteins, some of which are probably not toxins, were also identified in the proteomes and complement the venom composition. Although we expected great differences in the composition of both venoms due to dietary and geographic divergence between them, the general profiles were in fact quite similar between them. Differences, however, exist at the amino acid level between the versions of the proteins in each of these species and for other species of the genus previously investigated, indicating that this could be the basis of the poor recognition of *T*. *obscurus* venom by the antiserum compared to other species. The high abundance of a simplified form of ADAM-like metalloproteinases in either the transcriptomes and in the venom proteomes seems to be a rule in the genus, reinforcing the importance of understating the biological role of this component as well as its contribution to the envenoming syndrome.

## 4. Methods

### Biological samples

*Tityus obscurus* specimens were captured in the region between Belterra and Santarém municipalities in Pará state (3°10'18.95"S; 55° 1'8.57"O) and *Tityus serrulatus* specimens were captured in Altair municipality in São Paulo state (20°26'42.22"S; 49° 6'7.59"O). The genetic material was accessed under license of the Conselho de Gestão do Patrimônio Genético (CGEN, license # 010803/2013-0) and maintained at the Arthropod Laboratory of the Butantan Institute. The *Tityus serrulatus* venom was supplied by the Venom Commission of the Butantan Institute. Both venoms were collected by electrical stimulation of the telson of mature scorpions (a pool of 80 *Tityus obscurus* individuals and a pool of more than a hundred individuals of *Tityus serrulatus*) and were lyophilized immediately after extraction and kept at –20 °C. Before use, the venom was dissolved as described by [87]. Briefly, 1.5 mg of each venom was dissolved in 0.2 mL of ultrapure water, centrifuged at 10.015 g, 4 °C for 10 minutes. The precipitate was resuspended twice under the same conditions and the supernatants were pooled, resulting in the crude soluble venom without mucus. Protein concentration was determined by the Bradford assay using bovine albumin as standard and the solutions were kept at −20 °C.

### mRNA sequencing

Six telsons were pooled from males and females of *Tityus obscurus* individuals and fifteen telsons were pooled from females of *T*. *serrulatus*, 48 hours after being milked by electrical stimulation. The protocol followed the same methodologies that our group used for *T*. *bahiensis* [43]. For total RNA isolation, the telsons were ground into a powder in liquid nitrogen and homogenized in Polytron^®^ Tissue Homogenizer. Total RNA was extracted with TRIZOL Reagent (Invitrogen, Life Technologies Corp.) and mRNA was prepared with magnetic beads with an oligo (dT) using Dynabeads^®^ mRNA DIRECT kit (Invitrogen, Life Technologies Corp., Carlsbad, CA, US). mRNA was quantified by Quant-iT^™^ RiboGreen^®^ RNA reagent and Kit (Invitrogen, Life Technologies Corp.). Integrity of mRNA was evaluated in a 2100 Bioanalyzer, picochip series (Agilent Technologies Inc., Santa Clara, CA, US). Five hundred nanograms of mRNA were used for fragmentation using ZnCl_2_ solution at 70 °C for 30 seconds. Random primers were used to synthesize the first strand of cDNA using a standard cDNA Synthesis System (Roche Diagnostics). The cDNA was then subjected to fragment end-repair followed by adaptor ligation using a cDNA Rapid Library Prep kit (Roche Diagnostics). Purification of the cDNA fragments was carried out with Agencourt AMPure XP beads (Beckman Coulter Inc.). Emulsion PCR amplification of the cDNA library was performed according to the manufacturer’s instructions applying two molecules of cDNA per bead (Roche Diagnostics). Beads with clonally amplified cDNA library were selected and deposited in a picotiter plate for pyrosequencing using Titanium Sequencing Chemistry (Roche Diagnostics) with 200 flow cycles, in a GS Junior 454 Sequencing System (Roche Diagnostics), following the manufacturer protocols.

### Bioinformatic analysis

The analysis followed the same pipeline that our group used for *T*. *bahiensis* [43]. The total read dataset was used to construct a consensus *de novo* assembly with the Newbler v2.7 GS Assembler (Roche, Diagnostics, Indianapolis, IN, US) using the “cDNA option”. Ribosomal RNA sequences from Arachnids were downloaded from the GenBank and reads mapping to the rRNA were excluded from the assembly by using the filter option during assembly. A Newbler assembler also removed adaptors in the first step. A minimum overlap length of 95% of the read and a minimum overlap identity of 90% were set, with the other parameters set as the software default. Assembled isotigs were subjected to a Blast search against GenBank (NR and TSA database) and UniProt database with the alignment tool BlastX (E-value < 10^−6^) to identify similar sequences. The assembled sequences were automatically annotated using Blast2Go [55] using the default parameter settings to assign gene ontology terms (molecular function, cellular component, biological process) to each sequence. Toxin categories were attributed manually based on Blast best hits. Final manual curation of relevant isotig sequences was undertaken to improve the quality and to extend some of the assembled cDNAs.

The raw data generated in this project was deposited in the GenBank BioProject section under the accession code PRJNA260533 and BioSample SAMN03381142 and SAMN04563605. This Transcriptome Shotgun Assembly project was deposited at DDBJ/EMBL/GenBank under the accession GEMQ00000000 and GEUW00000000. The version described in this paper is the first version, GEMQ01000000 and GEUW01000000.

Expression values were accessed by the RPKM (reads per kilobase per million mapped reads), calculated by using RNA-Seq function of CLC Genomics Workbench 5.5.1 software. The nucleotide sequences of each individual toxin were translated into amino acid sequences and aligned by ClustalW [88] using default parameters, manually edited using Seaview [89] and, for presenting figures Boxshade (http://www.ch.embnet.org/software/BOX_form.html) was used. The identity percentages were calculated using SIAS server (http://imed.med.ucm.es/Tools/sias.html).

### RNA-seq database

To assist in the identification of potential coding regions within reconstructed transcripts, a TransDecoder software, version 2.0.1 (http://transdecoder.sourceforge.net/), was used with minimum protein length of 20. The transcript containing the coding candidate sequences were aligned by BLASTp [90] against the database Uniprot/Swissprot proteins and non-redundant (NR) NCBI to assess the protein description with cutoff value of 1e-05, and according to the criterion with longer protein similarity. The analysis of PFAM domains retained for the assembled and annotated proteins were identified with a hmmsearch tool in the software package hmmer3 [91], against a PFAM domains database [92]. The TransDecoder may predict more than one coding sequence candidate by transcript and only one candidate per transcript was selected, and the priority order of a UniPro- tKB/TrEMBL, Pfam database and NR-NCBI was used for annotating and selecting the best candidate for each transcript.

### Proteomic approach

The analyses were performed on a LTQ-Orbitrap Velos ETD (Thermo Fisher Scientific Inc. Waltham, MA, USA) coupled with Easy nanoLC II (Thermo Fisher). The peptides were separated on a C18RP column on a 70-minute gradient. The instrumental conditions were checked using 50-fmol of a tryptic digest of a BSA as standard. Briefly, 10μl of sample were injected into a Thermo Easy-nLC Velos with a C18 reverse phase column. A linear gradient from 1 to 95% solvent B was performed over 77 minutes at flow rate 300 nL/min, where solvent A was 0.1% formic acid and solvent B was 0.1% formic acid in acetonitrile. The other chromatography parameters used in the analysis of peptides were detailed in supplementary material S6.

Analyses of enzyme-digested samples were performed in a LTQ-Orbitrap Velos mass spectrometer (Thermo Scientific, Bremen, GA, USA) coupled to an Easy-nLC II (Thermo Fisher Scientific, Bremen, GA, USA). The mass spectrometer was operated in DDA mode in which full MS scan was acquired in the *m*/*z* range of 100–1300 followed by MS/MS acquisition using high collision dissociation (HCD) of the seven most intense ions from the MS scan. MS spectra were acquired in the Orbitrap analyzer at 60,000 resolution (at 400 *m*/*z*) whereas the MS/MS scans were acquired in the linear ion trap. The isolation window for precursor ions was set to 2 *m/z*, the minimum count to trigger events (MS^2^) was 15,000 cps. Normalized collision energy was set to 35%. Enzyme-digested samples were analyzed in duplicate. The other MS/MS parameters used in the acquisition of peptides were detailed in supplementary material S1 Methods.

The raw data from MS/MS were converted using the MSconvert software, version 3.0.6398 [93] into mgf a mascot generic file. We merged the output files with the two technical replicates. The mgf file and the predicted database were used in Mascot (Matrix Science, London, UK; available at: http://www.matrixscience.com) search. Mascot search were set up to search peptides in the predicted databases (3591 and 3977 sequences from *T*. *obscurus* and *T serrulatus*, respectively), combined with 245 sequences of common contaminants. A reverse version of all sequences (decoy) was also included in the database. Enzyme specificity was set to trypsin and at least two missed cleavages were allowed. A false discovery rate (FDR) of 1.0%, *p-value* < 0.01 and *e-value* < 0.05 were required for identifications, the score values were also observed. The identified isotigs were selected and grouped as proteins with peptide evidence. The remaining Mascot search parameters used in the analysis of peptides were detailed on supplementary material S1 Methods.

### Phylogenetic analysis

We selected the mature protein sequences of the putative metalloproteinases without the signal peptide to be used in Prottest 2.4 [94]. Prottest selected the model of protein evolution that best fit in the sequence alignment; WAG with site heterogeneity model gamma plus invariant sites (G+*I*). The Bayesian analyses were carried out using Markov chain Monte Carlo (MCMC) implemented in BEAST 1.7.5 package [95]. We ran four independent MCMC searches using distinct randomly generated starting trees. Each run consisted of 50-million generations, and trees were sampled every 1,000 generations. Convergence was inspected in Tracer v1.5 [95]. All runs reached a stationary level after 10% BurnIn with a large effective sample size. Trees obtained after the BurnIn step were used to generate a maximum clade credibility tree with TreeAnnotator v1.7.5 [95], using a majority rule. The resulting tree was visualized and edited using FigTree v1.4.0 (unpublished, available at http://tree.bio.ed.ac.uk/software/figtree).

### SDS-PAGE and western blotting analysis

This assay was performed with venom from *Tityus serrulatus* and *Tityus obscurus*. The proteins of the venoms (30 μg of protein) were denatured in sample buffer [96] for 5 minutes at 100°C and separated by SDS-polyacrylamide gel (running gel 12%) electrophoresis. The gel was stained with Coomassie Brilliant Blue R or the proteins were electrophoretically transferred onto nitrocellulose membranes. Nonspecific binding sites were blocked with 5% nonfat milk in PBS for 1 hour at room temperature. Membranes were then incubated with horse anti- *Tityus serrulatus* venom serum (1:1000) produced by the Instituto Butantan for therapeutic use, composed by F(ab’)2 immunoglobulin fragments capable of neutralizing at least 7.5 MLD (Minimum Lethal Dose in guinea-pigs) of reference venom of *Tityus serrulatus* for 2 hours at room temperature. After washing with PBS containing 0.2% tween-20, the membranes were probed with HRP-conjugated secondary antibodies (1:10.000, Sigma-Aldrich, St. Louis, MO USA) for 30 minutes at room temperature. Immunoreactive protein bands were visualized using an enhanced chemiluminescence detection system (SuperSignal West Pico Substrate, Thermo Fisher Scientific, Bremen, Germany). Protein bands were detected with a ChemiDoc XRS photodocumentation system using Quantity One software (Bio-Rad, Hercules, CA).

## Supporting information

S1 Table Table describing the results of Newbler assembled sequences for *T*. *obscurus* and *T*. *serrulatus*. (PDF)

S2 Table Annotation table describing the putative venom components, RPKM and blastX results from *T*. *obscurus*. (XLSX)

S3 Table Annotation table describing the putative venom components, RPKM and blastX results from *T*. *serrulatus*. (XLSX)

S4 Table Table describing the peptides detected by MS/MS and mapping to transcripts, from *T*. *obscurus*. (XLSX)

S5 Table Table describing the peptides detected by MS/MS and mapping to transcripts, from *T*. *serrulatus*. (XLSX)

S1 Fig *Tityus obscurus* isotig expression according to the isotig lengths. Isotigs annotated as cellular components and putative venom components. The RPKM values are represented by blue bars and refer to the scale on the left axis. The reads per isotig values are represented by red bars and refer to the scale on the right axis. Isotig lengths are indicated by the brown line and refers to the scale in the left axis.
(TIF)

S2 Fig *Tityus serrulatus* isotig expression according to the isotig lengths. Isotigs annotated as cellular components and putative venom components. The RPKM values are represented by blue bars and refer to the scale on the left axis. The reads per isotig values are represented by red bars and refer to the scale on the right axis. Isotig lengths are indicated by the brown line and refers to the scale in the left axis.
(TIF)

S3 Fig Alignment of the amino acid sequences of putative metalloproteinase domains from *T*. *obscurus* (Tobs) and *T*. *serrulatus* (Tser), other scorpions, arachnids and vertebrates metalloproteinase. Variations in gray scale indicate levels of sequence conservation. The percentages of identity compared to the top sequence are indicated at the end of the alignment. The symbol (-) represents gaps to improve the alignment. A pink line indicates the metal binding site. NP055080—ADAM 28 isoform 1 *Homo sapiens*, XP001233496—ADAM 28 isoform X1 *Gallus gallus*, Q5XUW8—Snake venom metalloproteinase insularinase-A, Q90392—Snake venom metalloproteinase atrolysin-C *Crotalus atrox*, BAE72663—metalloproteinase partial from *Haemaphysalis longicornis*, AAZ39661—salivary gland metalloproteinase *Rhipicephalus microplus*, JAA93001—putative ADAMTS *Cupiennius salei*, JAB68845—putative ADAMTS 7 *Ixodes ricinus*, ABR20110—venom metalloprotease-1 *Mesobuthus eupeus*, ABR20111—venom metalloprotease-2 *Mesobuthus eupeus*, P86392—venom metalloproteinase antarease from *T*. *serrulatus*, P85842—venom metalloproteinases from *T*. *serrulatus*, A0A076L876, A0A076LAV6, A0A076LAV7, A0A076L316, A0A076L339, A0A076L882, A0A076L7Z5, A0A076L3I0 and A0A076L332—*metalloserrulases from T*. serrulattus, V9Z9A3- venom metalloproteinase antarease-like from *T*. *serrulatus*, V9Z548 and V9ZAX6—Venom metalloproteinase antarease-like from *T*. *pachyurus*, V9ZAY0—Venom metalloproteinase antarease-like from *T*. *trivittatus*, V9Z7R6—Venom metalloproteinase antarease-like from *T*. *fasciolatus*, JK483842, JK483742, JK483774—are *Tityus stigmurus* similar to antarease, AHE40588 and AHE40589 are *T*. *serrulatus* antarease-like, JAG85190, JAG85190 and JAG85200 are putative venom metalloproteinase from *T*. *bahiensis*.
(TIF)

S1 Methods Material and methods used on proteomic approach for both species: Chromatographic conditions, MS/MS detection parameters and Mascot search parameters. (DOCX)

## Abbreviations

ACE-like: angiotensin converting enzyme-like
ADAM: *A Disintegrin And Metalloproteinase*
ADAM-TS: A Disintegrin And Metalloproteinase with Thrombospondin Motifs
BPP: Bradykinin-potentiating peptide
CRISP: Cystein-rich secretory protein
ECE-like: endothelin converting enzyme-like
PLA2: Phospholipase A2
PLC: Phospholipase C
*T. bahiensis*: *Tityus bahiensis*
*T. obscurus*: *Tityus obscurus*
*T. serrulatus*: *Tityus serrulatus*

## References

[1] Fet V, Sissom W.D., Lowe G. & Braunwalder M. E (2000) Catalog of the Scorpions of the World (1758–1998). New York: New York Entomological Society.

[2] Reckziegel GC, Pinto VL Jr. (2014) Scorpionism in Brazil in the years 2000 to 2012. J Venom Anim Toxins Incl Trop Dis 20: 46 doi: 10.1186/1678-9199-20-46 25873937 10.1186/1678-9199-20-46 PMC4396563

[3] Chippaux JP (2015) Epidemiology of envenomations by terrestrial venomous animals in Brazil based on case reporting: from obvious facts to contingencies. J Venom Anim Toxins Incl Trop Dis 21: 13 doi: 10.1186/s40409-015-0011-1 26042152 10.1186/s40409-015-0011-1 PMC4453217

[4] YAMANO EYSH A. S. V.; NEGRÃO S. G.; SOUZA N.; LIMA S. G. L.; SOUZA Z. N.; MAGALHÃES A. A.; MIRANDA J. B. B.; ESTEVES F. A. L.; VIEIRA J. L.; PARDAL P. P. (1999) Aspectos epidemiológicos e Clínicos dos acidentes por escorpiões orientados pelo Centro de Informações Toxicológicas de Belém, no período de maio de 1997 a novembro de 1998. Revista da Sociedade Brasileira de Medicina Tropical 32.

[5] Sinan (2015) Scorpions accidents in Brazil 2013–2015. Ministério da Saúde/SVS—Sistema de Informação de Agravos de Notificação—Sinan Net.

[6] Chippaux JP, Goyffon M (2008) Epidemiology of scorpionism: a global appraisal. Acta Trop 107: 71–79. doi: 10.1016/j.actatropica.2008.05.021 18579104 10.1016/j.actatropica.2008.05.021

[7] Lourenço WR, Leguin EA (2008) The true identity of *Scorpio* (Atreus) obscurus Gervais, 1843 (Scorpiones, Buthidae) In: V F, editor. Euscorpius—Occasional Publications in Scorpiology. Huntington, WV: Marshall University pp. 1–11.

[8] Lourenco WR (2015) What do we know about some of the most conspicuous scorpion species of the genus *Tityus*? A historical approach. J Venom Anim Toxins Incl Trop Dis 21: 20 doi: 10.1186/s40409-015-0016-9 26085830 10.1186/s40409-015-0016-9 PMC4470017

[9] Pardal PP, Ishikawa EA, Vieira JL, Coelho JS, Dorea RC, et al (2014) Clinical aspects of envenomation caused by *Tityus obscurus* (Gervais, 1843) in two distinct regions of Para state, Brazilian Amazon basin: a prospective case series. J Venom Anim Toxins Incl Trop Dis 20: 3 doi: 10.1186/1678-9199-20-3 24517181 10.1186/1678-9199-20-3 PMC3923241

[10] Pardal PP, Castro LC, Jennings E, Pardal JS, Monteiro MR (2003) [Epidemiological and clinical aspects of scorpion envenomation in the region of Santarem, Para, Brazil]. Rev Soc Bras Med Trop 36: 349–353. 12908035 10.1590/s0037-86822003000300006

[11] Torrez PPQ, Quiroga MMM, Abati PAM, Mascheretti M, Costa WS, et al (2015) Acute cerebellar dysfunction with neuromuscular manifestations after scorpionism presumably caused by *Tityus obscurus* in Santarem, Para/Brazil. Toxicon 96: 68–73. doi: 10.1016/j.toxicon.2014.12.012 25549940 10.1016/j.toxicon.2014.12.012

[12] Cologna CT, Marcussi S, Giglio JR, Soares AM, Arantes EC (2009) *Tityus serrulatus* scorpion venom and toxins: an overview. Protein Pept Lett 16: 920–932. 19689419 10.2174/092986609788923329

[13] Pucca MB, Cerni FA, Pinheiro EL Junior, Bordon Kde C, Amorim FG, et al (2015) *Tityus serrulatus* venom—A lethal cocktail. Toxicon 108: 272–284. doi: 10.1016/j.toxicon.2015.10.015 26522893 10.1016/j.toxicon.2015.10.015

[14] Quintero-Hernandez V, Jimenez-Vargas JM, Gurrola GB, Valdivia HH, Possani LD (2013) Scorpion venom components that affect ion-channels function. Toxicon 76: 328–342. doi: 10.1016/j.toxicon.2013.07.012 23891887 10.1016/j.toxicon.2013.07.012 PMC4089097

[15] Gurevitz M, Karbat I, Cohen L, Ilan N, Kahn R, et al (2007) The insecticidal potential of scorpion beta-toxins. Toxicon 49: 473–489. doi: 10.1016/j.toxicon.2006.11.015 17197009 10.1016/j.toxicon.2006.11.015

[16] Campos FV, Chanda B, Beirao PSL, Bezanilla F (2008) alpha-scorpion toxin impairs a conformational change that leads to fast inactivation of muscle sodium channels. Journal of General Physiology 132: 251–263. doi: 10.1085/jgp.200809995 18663133 10.1085/jgp.200809995 PMC2483334

[17] Guerrero-Vargas JA, Mourao CB, Quintero-Hernandez V, Possani LD, Schwartz EF (2012) Identification and phylogenetic analysis of *Tityus pachyurus* and *Tityus obscurus* novel putative Na+-channel scorpion toxins. PLoS One 7: e30478 doi: 10.1371/journal.pone.0030478 22355312 10.1371/journal.pone.0030478 PMC3280238

[18] Martin-Eauclaire MF, Pimenta AM, Bougis PE, De Lima ME (2016) Potassium channel blockers from the venom of the Brazilian scorpion *Tityus serrulatus* (). Toxicon 119: 253–265. doi: 10.1016/j.toxicon.2016.06.016 27349167 10.1016/j.toxicon.2016.06.016

[19] Possani LD, Becerril B, Delepierre M, Tytgat J (1999) Scorpion toxins specific for Na+-channels. European Journal of Biochemistry 264: 287–300. 10491073 10.1046/j.1432-1327.1999.00625.x

[20] Verano-Braga T, Rocha-Resende C, Silva DM, Ianzer D, Martin-Eauclaire MF, et al (2008) *Tityus serrulatus* Hypotensins: a new family of peptides from scorpion venom. Biochem Biophys Res Commun 371: 515–520. doi: 10.1016/j.bbrc.2008.04.104 18445483 10.1016/j.bbrc.2008.04.104

[21] Guo X, Ma C, Du Q, Wei R, Wang L, et al (2013) Two peptides, TsAP-1 and TsAP-2, from the venom of the Brazilian yellow scorpion, T*ityus serrulatus*: evaluation of their antimicrobial and anticancer activities. Biochimie 95: 1784–1794. doi: 10.1016/j.biochi.2013.06.003 23770440 10.1016/j.biochi.2013.06.003

[22] Ferreira LA, Henriques OB (1992) Isolation of a bradykinin-potentiating factor from scorpion *Tityus serrulatus* venom. Agents Actions Suppl 38 (Pt 1): 462–468. 10.1007/978-3-0348-7321-5_58 1466294

[23] Pessini AC, Takao TT, Cavalheiro EC, Vichnewski W, Sampaio SV, et al (2001) A hyaluronidase from *Tityus serrulatus* scorpion venom: isolation, characterization and inhibition by flavonoids. Toxicon 39: 1495–1504. 11478957 10.1016/s0041-0101(01)00122-2

[24] Almeida FM, Pimenta AM, De Figueiredo SG, Santoro MM, Martin-Eauclaire MF, et al (2002) Enzymes with gelatinolytic activity can be found in *Tityus bahiensis* and *Tityus serrulatu*s venoms. Toxicon 40: 1041–1045. 12076659 10.1016/s0041-0101(02)00084-3

[25] Fletcher PL Jr., Fletcher MD, Weninger K, Anderson TE, Martin BM (2010) Vesicle-associated membrane protein (VAMP) cleavage by a new metalloprotease from the Brazilian scorpion *Tityus serrulatus*. J Biol Chem 285: 7405–7416. doi: 10.1074/jbc.M109.028365 20026600 10.1074/jbc.M109.028365 PMC2844189

[26] Carmo AO, Oliveira-Mendes BB, Horta CC, Magalhaes BF, Dantas AE, et al (2014) Molecular and functional characterization of metalloserrulases, new metalloproteases from the *Tityus serrulatus* venom gland. Toxicon 90: 45–55. doi: 10.1016/j.toxicon.2014.07.014 25091350 10.1016/j.toxicon.2014.07.014

[27] Pucca MB, Cerni FA, Pinheiro EL Junior, Zoccal KF, Bordon Kde C, et al (2016) Non-disulfide-bridged peptides from Tityus serrulatus venom: Evidence for proline-free ACE-inhibitors. Peptides 82: 44–51. doi: 10.1016/j.peptides.2016.05.008 27221550 10.1016/j.peptides.2016.05.008

[28] Kalapothakis E, Jardim S, Magalhaes AC, Mendes TM, De Marco L, et al (2001) Screening of expression libraries using ELISA: identification of immunogenic proteins from *Tityus bahiensis* and *Tityus serrulatus* venom. Toxicon 39: 679–685. 11072047 10.1016/s0041-0101(00)00194-x

[29] Pimenta AM, Stocklin R, Favreau P, Bougis PE, Martin-Eauclaire MF (2001) Moving pieces in a proteomic puzzle: mass fingerprinting of toxic fractions from the venom of *Tityus serrulatus* (Scorpiones, Buthidae). Rapid Commun Mass Spectrom 15: 1562–1572. doi: 10.1002/rcm.415 11713783 10.1002/rcm.415

[30] Alvarenga ER, Mendes TM, Magalhaes BF, Siqueira FF, Dantas AE, et al (2012) Transcriptome analysis of the *Tityus serrulatus* scorpion venom gland. Open Journal of Genetics 02: 210–220.

[31] Batista CV, Gomez-Lagunas F, Lucas S, Possani LD (2000) Tc1, from *Tityus cambridgei*, is the first member of a new subfamily of scorpion toxin that blocks K(+)-channels. FEBS Lett 486: 117–120. 11113450 10.1016/s0014-5793(00)02253-5

[32] Batista CV, Zamudio FZ, Lucas S, Fox JW, Frau A, et al (2002) Scorpion toxins from *Tityus cambridgei* that affect Na(+)-channels. Toxicon 40: 557–562. 11821128 10.1016/s0041-0101(01)00252-5

[33] Batista CV, del Pozo L, Zamudio FZ, Contreras S, Becerril B, et al (2004) Proteomics of the venom from the Amazonian scorpion *Tityus cambridgei* and the role of prolines on mass spectrometry analysis of toxins. J Chromatogr B Analyt Technol Biomed Life Sci 803: 55–66. doi: 10.1016/j.jchromb.2003.09.002 15025998 10.1016/j.jchromb.2003.09.002

[34] Schwartz EF, Diego-Garcia E, de la Vega RCR, Possani LD (2007) Transcriptome analysis of the venom gland of the Mexican scorpion *Hadrurus gertschi* (Arachnida: Scorpiones). Bmc Genomics 8. 10.1186/1471-2164-8-119 PMC1904202 17506894

[35] Ma Y, Zhao R, He Y, Li S, Liu J, et al (2009) Transcriptome analysis of the venom gland of the scorpion *Scorpiops jendeki*: implication for the evolution of the scorpion venom arsenal. BMC Genomics 10: 290 doi: 10.1186/1471-2164-10-290 19570192 10.1186/1471-2164-10-290 PMC2713264

[36] Ruiming Z, Yibao M, Yawen H, Zhiyong D, Yingliang W, et al (2010) Comparative venom gland transcriptome analysis of the scorpion *Lychas mucronatus* reveals intraspecific toxic gene diversity and new venomous components. BMC Genomics 11: 452 doi: 10.1186/1471-2164-11-452 20663230 10.1186/1471-2164-11-452 PMC3091649

[37] Morgenstern D, Rohde BH, King GF, Tal T, Sher D, et al (2011) The tale of a resting gland: transcriptome of a replete venom gland from the scorpion *Hottentotta judaicus*. Toxicon 57: 695–703. doi: 10.1016/j.toxicon.2011.02.001 21329713 10.1016/j.toxicon.2011.02.001

[38] D'Suze G, Schwartz EF, Garcia-Gomez BI, Sevcik C, Possani LD (2009) Molecular cloning and nucleotide sequence analysis of genes from a cDNA library of the scorpion *Tityus discrepans*. Biochimie 91: 1010–1019. doi: 10.1016/j.biochi.2009.05.005 19470401 10.1016/j.biochi.2009.05.005

[39] Bringans S, Eriksen S, Kendrick T, Gopalakrishnakone P, Livk A, et al (2008) Proteomic analysis of the venom of *Heterometrus longimanus* (Asian black scorpion). Proteomics 8: 1081–1096. doi: 10.1002/pmic.200700948 18246572 10.1002/pmic.200700948

[40] Rates B, Ferraz KK, Borges MH, Richardson M, De Lima ME, et al (2008) *Tityus serrulatus* venom peptidomics: assessing venom peptide diversity. Toxicon 52: 611–618. doi: 10.1016/j.toxicon.2008.07.010 18718845 10.1016/j.toxicon.2008.07.010

[41] Ma YB, He YW, Zhao RM, Wu YL, Li WX, et al (2012) Extreme diversity of scorpion venom peptides and proteins revealed by transcriptomic analysis: Implication for proteome evolution of scorpion venom arsenal. Journal of Proteomics 75: 1563–1576. doi: 10.1016/j.jprot.2011.11.029 22155128 10.1016/j.jprot.2011.11.029

[42] Almeida DD, Scortecci KC, Kobashi LS, Agnez-Lima LF, Medeiros SR, et al (2012) Profiling the resting venom gland of the scorpion *Tityus stigmurus* through a transcriptomic survey. BMC Genomics 13: 362 doi: 10.1186/1471-2164-13-362 22853446 10.1186/1471-2164-13-362 PMC3444934

[43] de Oliveira UC, Candido DM, Dorce VA, Junqueira-de-Azevedo Ide L (2015) The transcriptome recipe for the venom cocktail of *Tityus bahiensis* scorpion. Toxicon 95: 52–61. doi: 10.1016/j.toxicon.2014.12.013 25553591 10.1016/j.toxicon.2014.12.013

[44] Luna-Ramirez K, Quintero-Hernandez V, Juarez-Gonzalez VR, Possani LD (2015) Whole Transcriptome of the Venom Gland from *Urodacus yaschenkoi* Scorpion. PLoS One 10: e0127883 doi: 10.1371/journal.pone.0127883 26020943 10.1371/journal.pone.0127883 PMC4447460

[45] Ma Y, Zhao Y, Zhao R, Zhang W, He Y, et al (2010) Molecular diversity of toxic components from the scorpion *Heterometrus petersii* venom revealed by proteomic and transcriptome analysis. Proteomics 10: 2471–2485. doi: 10.1002/pmic.200900763 20443192 10.1002/pmic.200900763

[46] Diego-Garcia E, Peigneur S, Clynen E, Marien T, Czech L, et al (2012) Molecular diversity of the telson and venom components from *Pandinus cavimanus* (Scorpionidae Latreille 1802): transcriptome, venomics and function. Proteomics 12: 313–328. doi: 10.1002/pmic.201100409 22121013 10.1002/pmic.201100409

[47] Abdel-Rahman MA, Quintero-Hernandez V, Possani LD (2013) Venom proteomic and venomous glands transcriptomic analysis of the Egyptian scorpion *Scorpio maurus* palmatus (Arachnida: Scorpionidae). Toxicon 74: 193–207. doi: 10.1016/j.toxicon.2013.08.064 23998939 10.1016/j.toxicon.2013.08.064

[48] Valdez-Velazquez LL, Quintero-Hernandez V, Romero-Gutierrez MT, Coronas FI, Possani LD (2013) Mass fingerprinting of the venom and transcriptome of venom gland of scorpion *Centruroides tecomanus*. PLoS One 8: e66486 doi: 10.1371/journal.pone.0066486 23840487 10.1371/journal.pone.0066486 PMC3688770

[49] Zhang L, Shi W, Zeng XC, Ge F, Yang M, et al (2015) Unique diversity of the venom peptides from the scorpion *Androctonus bicolor* revealed by transcriptomic and proteomic analysis. J Proteomics 128: 231–250. doi: 10.1016/j.jprot.2015.07.030 26254009 10.1016/j.jprot.2015.07.030

[50] Rokyta DR, Ward MJ (2017) Venom-gland transcriptomics and venom proteomics of the black-back scorpion (*Hadrurus spadix*) reveal detectability challenges and an unexplored realm of animal toxin diversity. Toxicon 128: 23–37. doi: 10.1016/j.toxicon.2017.01.014 28115184 10.1016/j.toxicon.2017.01.014

[51] Santibanez-Lopez CE, Cid-Uribe JI, Zamudio FZ, Batista CVF, Ortiz E, et al (2017) Venom gland transcriptomic and venom proteomic analyses of the scorpion *Megacormus gertschi* Diaz-Najera, 1966 (Scorpiones: Euscorpiidae: Megacorminae). Toxicon 133: 95–109. doi: 10.1016/j.toxicon.2017.05.002 28478058 10.1016/j.toxicon.2017.05.002

[52] Santibanez-Lopez CE, Cid-Uribe JI, Batista CV, Ortiz E, Possani LD (2016) Venom Gland Transcriptomic and Proteomic Analyses of the Enigmatic Scorpion *Superstitionia donensis* (Scorpiones: Superstitioniidae), with Insights on the Evolution of Its Venom Components. Toxins (Basel) 8. 10.3390/toxins8120367 PMC5198561 27941686

[53] Kuzmenkov AI, Vassilevski AA, Kudryashova KS, Nekrasova OV, Peigneur S, et al (2015) Variability of Potassium Channel Blockers in *Mesobuthus eupeus* Scorpion Venom with Focus on Kv1.1: AN INTEGRATED TRANSCRIPTOMIC AND PROTEOMIC STUDY. J Biol Chem 290: 12195–12209. doi: 10.1074/jbc.M115.637611 25792741 10.1074/jbc.M115.637611 PMC4424352

[54] Xu XB, Duan ZG, Di ZY, He YW, Li JL, et al (2014) Proteomic analysis of the venom from the scorpion *Mesobuthus martensii*. Journal of Proteomics 106: 162–180. doi: 10.1016/j.jprot.2014.04.032 24780724 10.1016/j.jprot.2014.04.032

[55] Conesa A, Gotz S, Garcia-Gomez JM, Terol J, Talon M, et al (2005) Blast2GO: a universal tool for annotation, visualization and analysis in functional genomics research. Bioinformatics 21: 3674–3676. doi: 10.1093/bioinformatics/bti610 16081474 10.1093/bioinformatics/bti610

[56] Mortazavi A, Williams BA, McCue K, Schaeffer L, Wold B (2008) Mapping and quantifying mammalian transcriptomes by RNA-Seq. Nat Methods 5: 621–628. doi: 10.1038/nmeth.1226 18516045 10.1038/nmeth.1226 PMC13303166

[57] He Y, Zhao R, Di Z, Li Z, Xu X, et al (2013) Molecular diversity of *Chaerilidae* venom peptides reveals the dynamic evolution of scorpion venom components from *Buthidae* to non-*Buthidae*. J Proteomics 89: 1–14. doi: 10.1016/j.jprot.2013.06.007 23774330 10.1016/j.jprot.2013.06.007

[58] Alami M, Ouafik L, Ceard B, Legros C, Bougis PE, et al (2001) Characterisation of the gene encoding the alpha-toxin Amm V from the scorpion *Androctonus mauretanicus mauretanicus*. Toxicon 39: 1579–1585. 11478966 10.1016/s0041-0101(01)00140-4

[59] Zeng XC, Wang SX, Li WX (2002) Identification of BmKAPi, a novel type of scorpion venom peptide with peculiar disulfide bridge pattern from *Buthus martensii* Karsch. Toxicon 40: 1719–1722. 12457884 10.1016/s0041-0101(02)00134-4

[60] Rendon-Anaya M, Delaye L, Possani LD, Herrera-Estrella A (2012) Global transcriptome analysis of the scorpion *Centruroides noxius*: new toxin families and evolutionary insights from an ancestral scorpion species. PLoS One 7: e43331 doi: 10.1371/journal.pone.0043331 22912855 10.1371/journal.pone.0043331 PMC3422302

[61] Pimenta AM, Legros C, Almeida Fde M, Mansuelle P, De Lima ME, et al (2003) Novel structural class of four disulfide-bridged peptides from *Tityus serrulatus* venom. Biochem Biophys Res Commun 301: 1086–1092. 12589824 10.1016/s0006-291x(03)00082-2

[62] Batista CV, D'Suze G, Gomez-Lagunas F, Zamudio FZ, Encarnacion S, et al (2006) Proteomic analysis of *Tityus discrepans* scorpion venom and amino acid sequence of novel toxins. Proteomics 6: 3718–3727. doi: 10.1002/pmic.200500525 16705749 10.1002/pmic.200500525

[63] Batista CVF, Roman-Gonzalez SA, Salas-Castillo SP, Zamudio FZ, Gomez-Lagunas F, et al (2007) Proteomic analysis of the venom from the scorpion *Tityus stigmurus*: Biochemical and physiological comparison with other *Tityus* species. Comparative Biochemistry and Physiology C-Toxicology & Pharmacology 146: 147–157. 10.1016/j.cbpc.2006.12.004 17270501

[64] Schwartz EF, Camargos TS, Zamudio FZ, Silva LP, Bloch C, et al (2008) Mass spectrometry analysis, amino acid sequence and biological activity of venom components from the Brazilian scorpion *Opisthacanthus cayaporum*. Toxicon 51: 1499–1508. doi: 10.1016/j.toxicon.2008.03.029 18502464 10.1016/j.toxicon.2008.03.029

[65] Batista CVF, VillaHernandez O, Orihuela LH, Pando V, Possani LD (2009) Proteomic Analysis of the Venom from the Mexican Scorpion *Centruroides limpidus limpidus*. Molecular & Cellular Proteomics: S52–S52.

[66] Martin-Eauclaire MF, Granjeaud S, Belghazi M, Bougis PE (2013) Achieving automated scorpion venom mass fingerprinting (VMF) in the nanogram range. Toxicon 69: 211–218. doi: 10.1016/j.toxicon.2013.03.001 23500507 10.1016/j.toxicon.2013.03.001

[67] Verano-Braga T, Dutra AA, Leon IR, Melo-Braga MN, Roepstorff P, et al (2013) Moving pieces in a venomic puzzle: unveiling post-translationally modified toxins from *Tityus serrulatus*. J Proteome Res 12: 3460–3470. doi: 10.1021/pr4003068 23731212 10.1021/pr4003068

[68] Dias NB, de Souza BM, Cocchi FK, Chalkidis HM, Dorce VAC, et al (2018) Profiling the short, linear, non-disulfide bond-containing peptidome from the venom of the scorpion *Tityus obscurus*. J Proteomics 170: 70–79. doi: 10.1016/j.jprot.2017.09.006 28918200 10.1016/j.jprot.2017.09.006

[69] Alves RS, Ximenes RM, Jorge AR, Nascimento NR, Martins RD, et al (2013) Isolation, homology modeling and renal effects of a C-type natriuretic peptide from the venom of the Brazilian yellow scorpion (*Tityus serrulatus*). Toxicon 74: 19–26. doi: 10.1016/j.toxicon.2013.07.016 23911732 10.1016/j.toxicon.2013.07.016

[70] Horta CC, Magalhaes Bde F, Oliveira-Mendes BB, do Carmo AO, Duarte CG, et al (2014) Molecular, immunological, and biological characterization of *Tityus serrulatus* venom hyaluronidase: new insights into its role in envenomation. PLoS Negl Trop Dis 8: e2693 doi: 10.1371/journal.pntd.0002693 24551256 10.1371/journal.pntd.0002693 PMC3923731

[71] Duzzi B, Cajado-Carvalho D, Kuniyoshi AK, Kodama RT, Gozzo FC, et al (2016) [des-Arg(1)]-Proctolin: A novel NEP-like enzyme inhibitor identified in *Tityus serrulatus* venom. Peptides 80: 18–24. doi: 10.1016/j.peptides.2015.05.013 26056922 10.1016/j.peptides.2015.05.013

[72] Cajado-Carvalho D, Kuniyoshi AK, Duzzi B, Iwai LK, Oliveira UC, et al (2016) Insights into the Hypertensive Effects of *Tityus serrulatus* Scorpion Venom: Purification of an Angiotensin-Converting Enzyme-Like Peptidase. Toxins (Basel) 8. 10.3390/toxins8120348 PMC5198543 27886129

[73] Batista C, Zamudio F, Lucas S, Possani L. Abstract Tu-Po-14.; 1998; Margarita Island, Venezuela.

[74] Batista CV, Gomez-Lagunas F, Rodriguez de la Vega RC, Hajdu P, Panyi G, et al (2002) Two novel toxins from the Amazonian scorpion *Tityus cambridgei* that block Kv1.3 and Shaker B K(+)-channels with distinctly different affinities. Biochim Biophys Acta 1601: 123–131. 12445473 10.1016/s1570-9639(02)00458-2

[75] Murgia AR, Batista CVF, Prestipino G, Possani LD (2004) Amino acid sequence and function of a new alpha-toxin from the Amazonian scorpion *Tityus cambridgei*. Toxicon 43: 737–740. doi: 10.1016/j.toxicon.2004.02.014 15109895 10.1016/j.toxicon.2004.02.014

[76] Luna-Ramirez K, Quintero-Hernandez V, Vargas-Jaimes L, Batista CVF, Winkel KD, et al (2013) Characterization of the venom from the Australian scorpion *Urodacus yaschenkoi*: Molecular mass analysis of components, cDNA sequences and peptides with antimicrobial activity. Toxicon 63: 44–54. doi: 10.1016/j.toxicon.2012.11.017 23182832 10.1016/j.toxicon.2012.11.017

[77] de Paula Santos-da-Silva A, Candido DM, Nencioni AL, Kimura LF, Prezotto-Neto JP, et al (2017) Some pharmacological effects of *Tityus obscurus* venom in rats and mice. Toxicon 126: 51–58. doi: 10.1016/j.toxicon.2016.12.008 28012802 10.1016/j.toxicon.2016.12.008

[78] Markland FS Jr., Swenson S (2013) Snake venom metalloproteinases. Toxicon 62: 3–18. doi: 10.1016/j.toxicon.2012.09.004 23000249 10.1016/j.toxicon.2012.09.004

[79] Magalhães O (1946) Escorpionismo IV. Memórias do Instituto Oswaldo Cruz 3: 220.

[80] Possani LD, Martin BM, Fletcher MD, Fletcher PL (1991) Discharge Effect on Pancreatic Exocrine Secretion Produced by Toxins Purified from *Tityus serrulatus* Scorpion-Venom. Journal of Biological Chemistry 266: 3178–3185. 1993690

[81] Ortiz E, Rendon-Anaya M, Rego SC, Schwartz EF, Possani LD (2014) Antarease-like Zn-metalloproteases are ubiquitous in the venom of different scorpion genera. Biochim Biophys Acta 1840: 1738–1746. doi: 10.1016/j.bbagen.2013.12.012 24361608 10.1016/j.bbagen.2013.12.012

[82] Juarez P, Comas I, Gonzalez-Candelas F, Calvete JJ (2008) Evolution of Snake Venom Disintegrins by Positive Darwinian Selection. Molecular Biology and Evolution 25: 2391–2407. doi: 10.1093/molbev/msn179 18701431 10.1093/molbev/msn179

[83] Nishikawa AK, Caricati CP, Lima MLSR, Dossantos MC, Kipnis TL, et al (1994) Antigenic Cross-Reactivity among the Venoms from Several Species of Brazilian Scorpions. Toxicon 32: 989–998. 7985203 10.1016/0041-0101(94)90377-8

[84] Amaral CFS, Rezende NA (2000) Treatment of scorpion envenoming should include both a potent specific antivenom and support of vital functions. Toxicon 38: 1005–1007. 10836905 10.1016/s0041-0101(99)00158-0

[85] Cupo P (2015) Clinical update on scorpion envenoming. Rev Soc Bras Med Trop 48: 642–649. doi: 10.1590/0037-8682-0237-2015 26676487 10.1590/0037-8682-0237-2015

[86] Borges A, Rojas-Runjaic FJM, Diez N, Faks JG, den Camp HJMO, et al (2010) Envenomation by the Scorpion Tityus breweri in the Guayana Shield, Venezuela: Report of a Case, Efficacy and Reactivity of Antivenom, and Proposal for a Toxinological Partitioning of the Venezuelan Scorpion Fauna. Wilderness & Environmental Medicine 21: 282–290. 21168779 10.1016/j.wem.2010.06.008

[87] Pucca MB, Amorim FG, Cerni FA, Bordon KDF, Cardoso IA, et al (2014) Influence of post-starvation extraction time and prey-specific diet in *Tityus serrulatus* scorpion venom composition and hyaluronidase activity. Toxicon 90: 326–336. doi: 10.1016/j.toxicon.2014.08.064 25199494 10.1016/j.toxicon.2014.08.064

[88] Larkin MA, Blackshields G, Brown NP, Chenna R, McGettigan PA, et al (2007) Clustal W and Clustal X version 2.0. Bioinformatics 23: 2947–2948. doi: 10.1093/bioinformatics/btm404 17846036 10.1093/bioinformatics/btm404

[89] Gouy M, Guindon S, Gascuel O (2010) SeaView version 4: A multiplatform graphical user interface for sequence alignment and phylogenetic tree building. Mol Biol Evol 27: 221–224. doi: 10.1093/molbev/msp259 19854763 10.1093/molbev/msp259

[90] Altschul SF, Madden TL, Schaffer AA, Zhang J, Zhang Z, et al (1997) Gapped BLAST and PSI-BLAST: a new generation of protein database search programs. Nucleic Acids Res 25: 3389–3402. 9254694 10.1093/nar/25.17.3389 PMC146917

[91] Mistry J, Finn RD, Eddy SR, Bateman A, Punta M (2013) Challenges in homology search: HMMER3 and convergent evolution of coiled-coil regions. Nucleic Acids Res 41: e121 doi: 10.1093/nar/gkt263 23598997 10.1093/nar/gkt263 PMC3695513

[92] Bateman A, Coin L, Durbin R, Finn RD, Hollich V, et al (2004) The Pfam protein families database. Nucleic Acids Res 32: D138–141. doi: 10.1093/nar/gkh121 14681378 10.1093/nar/gkh121 PMC308855

[93] Kessner D, Chambers M, Burke R, Agus D, Mallick P (2008) ProteoWizard: open source software for rapid proteomics tools development. Bioinformatics 24: 2534–2536. doi: 10.1093/bioinformatics/btn323 18606607 10.1093/bioinformatics/btn323 PMC2732273

[94] Abascal F, Zardoya R, Posada D (2005) ProtTest: selection of best-fit models of protein evolution. Bioinformatics 21: 2104–2105. doi: 10.1093/bioinformatics/bti263 15647292 10.1093/bioinformatics/bti263

[95] Drummond AJ, Rambaut A (2007) BEAST: Bayesian evolutionary analysis by sampling trees. BMC Evol Biol 7: 214 doi: 10.1186/1471-2148-7-214 17996036 10.1186/1471-2148-7-214 PMC2247476

[96] Laemmli UK (1970) Cleavage of structural proteins during the assembly of the head of bacteriophage T4. Nature 227: 680–685. 5432063 10.1038/227680a0

