# Supplementary material for: Proteomic endorsed transcriptomic profiles of venom glands from Tityus obscurus and T. serrulatus scorpions
Source: PLoS One. 2018 Mar 21;13(3):e0193739. doi: 10.1371/journal.pone.0193739 (PMC5862453; doi:10.1371/journal.pone.0193739)
Supplement: S1 Table — (PDF) [file pone.0193739.s001.pdf]

Supplementary Table S1: Summary of Newbler assembly statistics.

Summary	number of sequences *T. obscurus*	number of sequences *T. serrulatus*
Total number of reads	156,700	230,172
Removed by quality or rRNA	54,272	64,526
Total number of trimmed and filter reads	102,428	165,646
Total assembled reads	91,508	150,656
Not assembled reads	47,494	51,539
Number of isotigs	4,223	5,274
Number of isotigs after curation	4,280	5,282
