# Supplementary figures and images for: Proteomic endorsed transcriptomic profiles of venom glands from Tityus obscurus and T. serrulatus scorpions
Source: PLoS One. 2018 Mar 21;13(3):e0193739. doi: 10.1371/journal.pone.0193739 (PMC5862453; doi:10.1371/journal.pone.0193739)

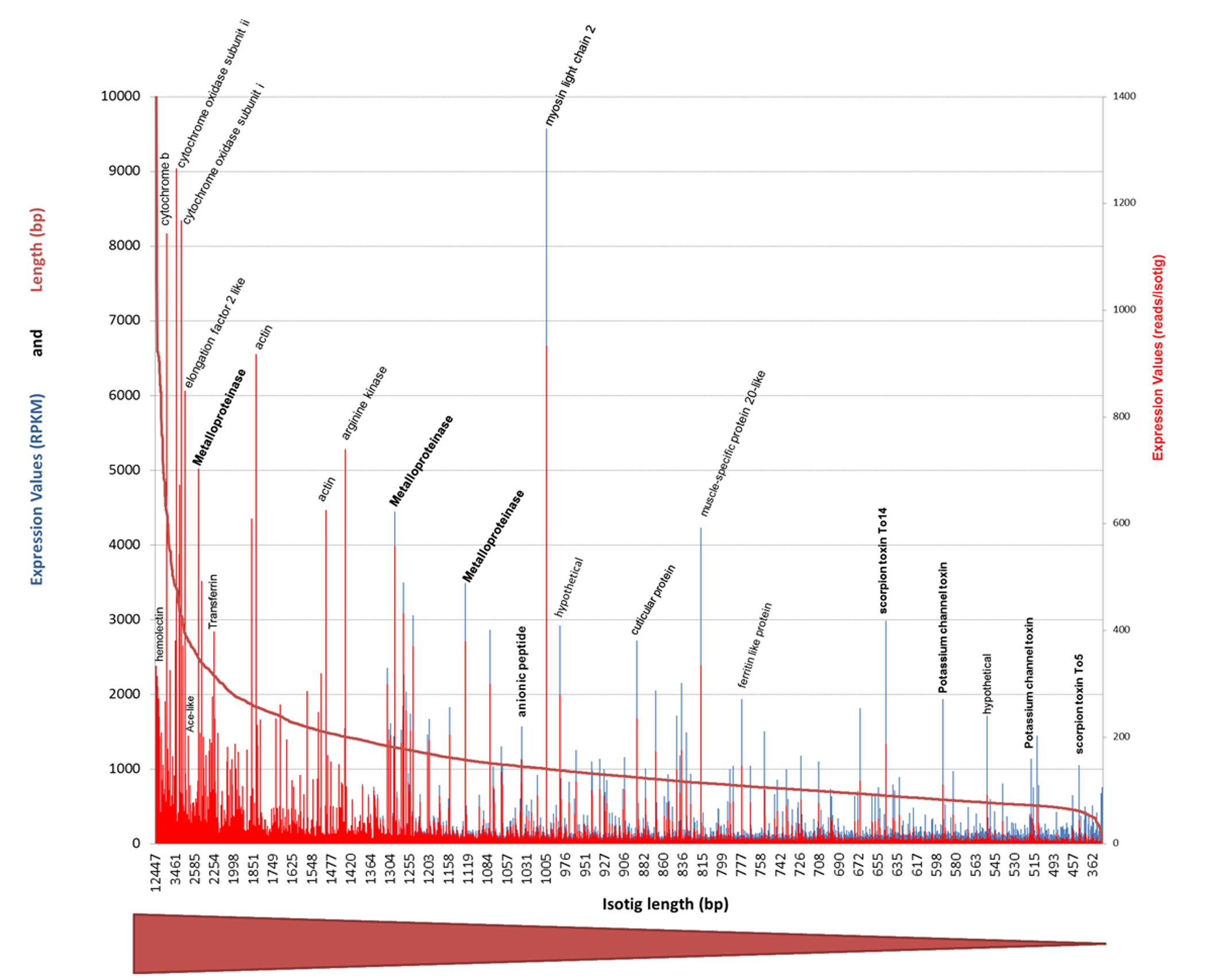

Supplement: S1 Fig — Isotigs annotated as cellular components and putative venom components. The RPKM values are represented by blue bars and refer to the scale on the left axis. The reads per isotig values are represented by red bars and refer to the scale on the right axis. Isotig lengths are indicated by the brown line and refers to the scale in the left axis. (TIF) [file pone.0193739.s006.tif]

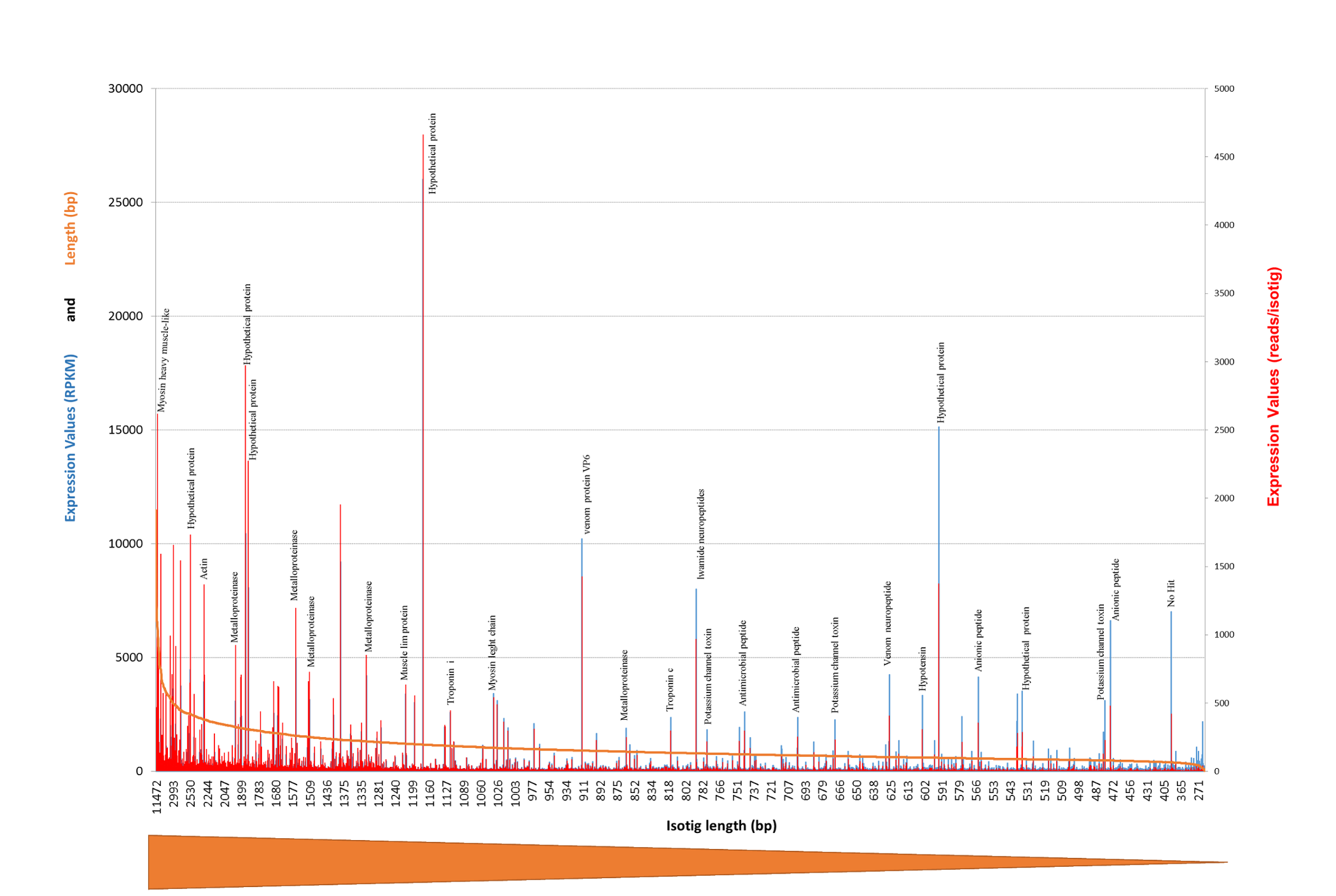

Supplement: S2 Fig — Isotigs annotated as cellular components and putative venom components. The RPKM values are represented by blue bars and refer to the scale on the left axis. The reads per isotig values are represented by red bars and refer to the scale on the right axis. Isotig lengths are indicated by the brown line and refers to the scale in the left axis. (TIF) [file pone.0193739.s007.tif]

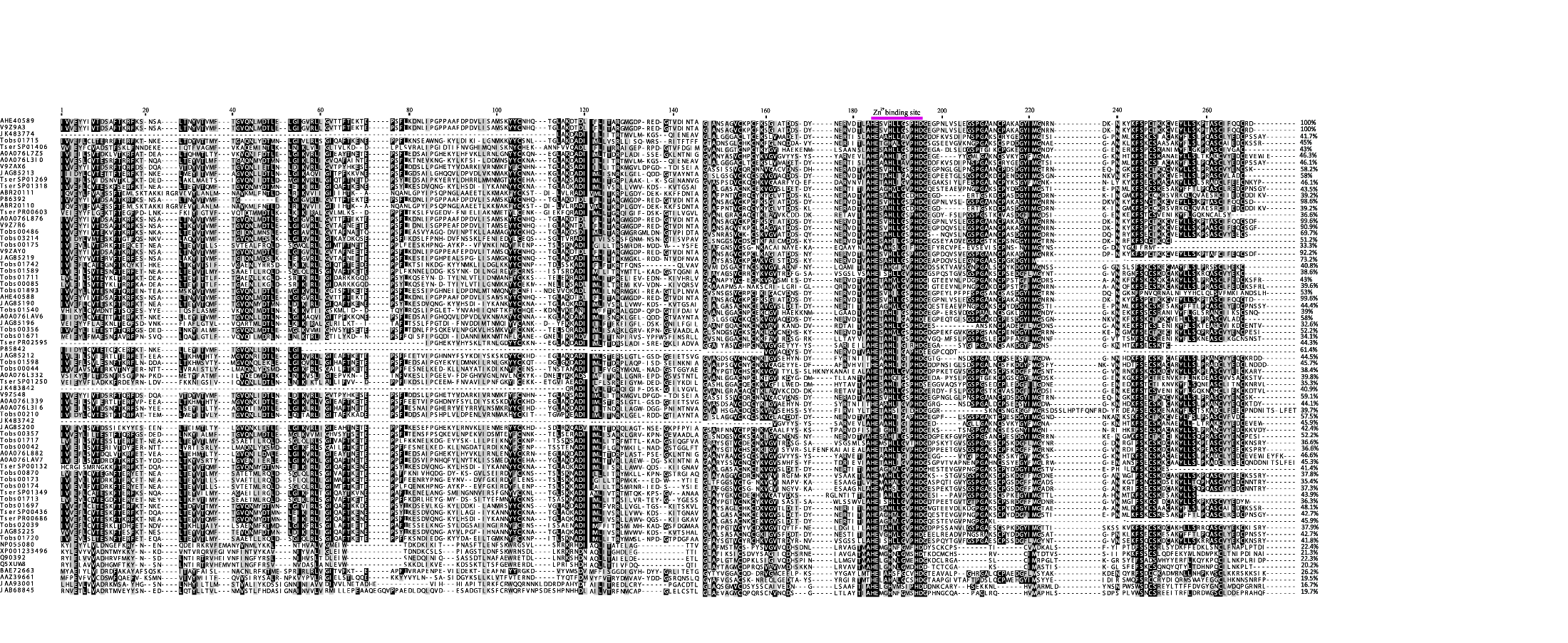

Supplement: S3 Fig — Variations in gray scale indicate levels of sequence conservation. The percentages of identity compared to the top sequence are indicated at the end of the alignment. The symbol (-) represents gaps to improve the alignment. A pink line indicates the metal binding site. NP055080—ADAM 28 isoform 1 Homo sapiens, XP001233496—ADAM 28 isoform X1 Gallus gallus, Q5XUW8—Snake venom metalloproteinase insularinase-A, Q90392—Snake venom metalloproteinase atrolysin-C Crotalus atrox, BAE72663—metalloproteinase partial from Haemaphysalis longicornis, AAZ39661—salivary gland metalloproteinase Rhipicephalus microplus, JAA93001—putative ADAMTS Cupiennius salei, JAB68845—putative ADAMTS 7 Ixodes ricinus, ABR20110—venom metalloprotease-1 Mesobuthus eupeus, ABR20111—venom metalloprotease-2 Mesobuthus eupeus, P86392—venom metalloproteinase antarease from T. serrulatus, P85842—venom metalloproteinases from T. serrulatus, A0A076L876, A0A076LAV6, A0A076LAV7, A0A076L316, A0A076L339, A0A076L882, A0A076L7Z5, A0A076L3I0 and A0A076L332—metalloserrulases from T. serrulattus, V9Z9A3- venom metalloproteinase antarease-like from T. serrulatus, V9Z548 and V9ZAX6—Venom metalloproteinase antarease-like from T. pachyurus, V9ZAY0—Venom metalloproteinase antarease-like from T. trivittatus, V9Z7R6—Venom metalloproteinase antarease-like from T. fasciolatus, JK483842, JK483742, JK483774—are Tityus stigmurus similar to antarease, AHE40588 and AHE40589 are T. serrulatus antarease-like, JAG85190, JAG85190 and JAG85200 are putative venom metalloproteinase from T. bahiensis. (TIF) [file pone.0193739.s008.tif]
