# Supplementary material for: Proteomic endorsed transcriptomic profiles of venom glands from Tityus obscurus and T. serrulatus scorpions
Source: PLoS One. 2018 Mar 21;13(3):e0193739. doi: 10.1371/journal.pone.0193739 (PMC5862453; doi:10.1371/journal.pone.0193739)
Supplement: S1 Methods — (DOCX) [file pone.0193739.s009.docx]

1. Chromatographic conditions for proteomic approach for *T. obscurus* and *T. serrulatus*

1.1 Thermo Easy-nLC method print for Velos

Sample pickup: Volume [µl]: 10.00 Flow [µl / min]: 20.00

Sample loading: Volume [µl]: 20.00 Flow [µl / min]: (unspecified) Max. Pressure [Bar]: 200.00

Gradient:

(A) 0.1% formic acid

(B) 0.1% formic acid in Acetonitrile

| Time [mm:ss] Duration [mm:ss] Flow [nl/min] M ixture [%B] |
| --- |
| 00:00 00:00 300.00 1.00 |
| 60:00 60:00 300.00 35.00 |
| 62:00 02:00 300.00 95.00 |
| 77:00 15:00 300.00 95.00 |

Pre-column equilibration: Volume [µl]: 12.00 Flow [µl / min]: (unspecified) Max. Pressure [Bar]: 200.00

Analytical column equilibration:

Volume [µl]: 3.00 Flow [µl / min]: (unspecified) Max. Pressure [Bar]: 200.00

Auto-sampler wash: Flush volume [µl]: 100.00

2. LTQ-Orbitrap MS/MS data acquisition parameters for proteomic analysis of *T. obscurus* and *T. serrulatus*

**2.1 LTQ Velos Instrument Method**

Creator: Velos

MS Run Time (min): 70.00

Sequence override of method parameters not enabled.

Divert Valve: not used during run

Contact Closure: not used during run

Syringe Pump: not used during run

MS Detector Settings:

Real-time modifications to method disabled

Stepped collision energy not enabled

Additional Microscans:

MS2 0 0

MS3 0 0

MS4 0 0

MS5 0 0

MS6 0 0

MS7 0 0

MS8 0 0

MS9 0 0

MS10 0 0

Experiment Type: Nth Order Double Play

Tune Method: Easy_nLC_Mar_26_2015_FGA

Scan Event Details:

1: FTMS + p norm ! 60000! pi o(100.0-1300.0)

CV = 0.0V

2: FTMS + p norm ! 60000! pi Dep MS/MS Most intense ion from (1)

Activation Type: HCD

Min. Signal Required: 15000.0

Isolation Width: 2.00

Normalized Coll. Energy: 35.0

Default Charge State: 2

Activation Time: 1.000

FT first mass mode: fixed at m/z

FT first mass value: 100.00

CV = 0.0V

Scan Event 2 repeated for top 7 peaks.

Lock Masses:

Pos List Name: N/A

Source: API Source

Mass List: (none)

Neg List Name: N/A

Source: API Source

Mass List: (none)

Data Dependent Settings:

Use separate polarity settings disabled

Parent Mass List: (none)

Reject Mass List: 144.99 149.02 157.03 205.08 223.06

228.93 235.20 249.18 279.16 297.08 323.91 341.95 355.07 385.93 401.92 429.09 445.12 519.14 593.16 593.16 667.17

Neutral Loss Mass List: (none)

Product Mass List: (none)

Neutral loss in top: 3

Product in top: 3

Most intense if no parent masses found not enabled

Add/subtract mass not enabled

FT master scan preview mode enabled

Charge state screening enabled

Charge state dependent ETD time not enabled

Monoisotopic precursor selection not enabled

Charge state rejection enabled

Unassigned charge states : rejected

Charge state 1 : rejected

Charge state 2 : not rejected

Charge state 3 : not rejected

Charge states 4+ : not rejected

Chromatography mode is disabled

Global Data Dependent Settings:

Predict ion injection time enabled

Use global parent and reject mass lists not enabled

Exclude parent mass from data dependent selection not enabled

Exclusion mass width by mass

Exclusion mass width low: 1.00

Exclusion mass width high: 1.50

Parent mass width by mass

Parent mass width low: 0.50

Parent mass width high: 0.50

Reject mass width relative to mass

Reject mass width relative to low (%): 0.00

Reject mass width relative to high (%): 0.00

Zoom/UltraZoom scan mass width by mass

Zoom/UltraZoom scan mass width low: 5.00

Zoom/UltraZoom scan mass width high: 5.00

FT SIM scan mass width low: 5.00

FT SIM scan mass width high: 5.00

Neutral Loss candidates processed by decreasing intensity

Neutral Loss mass width by mass

Neutral Loss mass width low: 0.50

Neutral Loss mass width high: 0.50

Product candidates processed by decreasing intensity

Product mass width by mass

Product mass width low: 0.50

Product mass width high: 0.50

MS mass range: 100.00-1300.00

MSn mass range by mass

MSn mass range: 0.00-1000000.00

Use m/z values as masses not enabled

Analog UV data dep. not enabled

Dynamic exclusion enabled

Repeat Count: 1

Repeat Duration: 30.00

Exclusion List Size: 500

Exclusion Duration: 15.00

Exclusion mass width by mass

Exclusion mass width low: 1.00

Exclusion mass width high: 1.50

Expiration: disabled

Isotopic data dependence not enabled

Custom Data Dependent Settings:

Not enabled

**3. Mascot search parameters for proteomic approach**

**3.1. *Tityus obscurus***

| **Search title** | ***Tityus obscurus*** |
| --- | --- |
| Peak list data path | Tobs_Merged.mgf |
| Peak list format | Mascot generic |
| Search type | MIS |
| Mascot version | 2.4.1 |
| Database | ScorpionTobsCon1217 |
| Fasta file | ScorpionTobsCon1217.fasta |
| Total sequences | 3591 |
| Total residues | 1073401 |
| Sequences after taxonomy filter | 3591 |
| Number of queries | 23885 |
|  |  |
| **Decoy** |  |
|  |  |
| Number of matches above identity threshold in search of real database | 682 |
| Number of matches above identity threshold in search of decoy database | 7 |
| Number of matches above homology threshold in search of real database | 682 |
| Number of matches above homology threshold in search of decoy database | 7 |
|  |  |
| **Fixed modifications** |  |
|  |  |
| Identifier | Name |
| 1 | Carbamidomethyl (C) |
| \| Delta \| \| --- \| \| 57.02146 \| | Neutral loss  0 |
|  |  |
| **Variable modifications** |  |
|  |  |
| Identifier | Name |
| 1 | Oxidation (M) |
| \| Delta \| \| --- \| \| 15.99492 \| | \| Neutral loss(es) \| \| \| --- \| --- \| \| 0 63.99829 \| |
|  |  |
| **Search Parameters** |  |
|  |  |
| Taxonomy filter | All entries |
| Enzyme | Trypsin |
| Maximum Missed Cleavages | 2 |
| Fixed modifications | Carbamidomethyl (C) |
| Quantitation method | None |
| Variable modifications | Oxidation (M) |
| Peptide Mass Tolerance | 10 |
| Peptide Mass Tolerance Units | ppm |
| Fragment Mass Tolerance | 0.5 |
| Fragment Mass Tolerance Units | Da |
| Mass values | Monoisotopic |
| Instrument type | ESI-TRAP |
| Isotope error mode | 0 |
| Decoy database also searched | 1 |
|  |  |
| **Format parameters** |  |
|  |  |
| Significance threshold | 0.011 |
| Max. number of hits | 0 |
| Use MudPIT protein scoring | 1 |
| Ions score cut-off | 0.05 |
| Include same-set proteins | 1 |
| Include sub-set proteins | 0 |
| Include unassigned | 0 |
| Require bold red | 0 |
| Use homology threshold | 0 |
| Group protein families | 0 |
| Re-score using Percolator | 0 |
|  |  |

**3.2. *Tityus serrulatus***

| **Search title** | ***Tityus serrulatus*** |
| --- | --- |
| Peak list data path | TserMerged.mgf |
| Peak list format | Mascot generic |
| Search type | MIS |
| Mascot version | 2.4.1 |
| Database | ScorpionTserCon1217 |
| Fasta file | ScorpionTserCon1217.fasta |
| Total sequences | 3977 |
| Total residues | 997325 |
| Sequences after taxonomy filter | 3977 |
| Number of queries | 30680 |
|  |  |
| **Decoy** |  |
|  |  |
| Number of matches above identity threshold in search of real database | 745 |
| Number of matches above identity threshold in search of decoy database | 7 |
| Number of matches above homology threshold in search of real database | 745 |
| Number of matches above homology threshold in search of decoy database | 7 |
|  |  |
| **Fixed modifications** |  |
|  |  |
| Identifier | Name |
| 1 | Carbamidomethyl (C) |
| \| Delta \| \| --- \| \| 57.021464 \| | Neutral loss  0 |
|  |  |
| **Variable modifications** |  |
|  |  |
| Identifier | Name |
| 1 | Oxidation (M) |
| \| Delta \|  \| \| \| --- \| --- \| --- \| \| 15.994915 \|  \|  \| | Neutral loss(es)  0 63.99829 |
|  |  |
| **Search Parameters** |  |
|  |  |
| Taxonomy filter | All entries |
| Enzyme | Trypsin |
| Maximum Missed Cleavages | 2 |
| Fixed modifications | Carbamidomethyl (C) |
| Quantitation method | None |
| Variable modifications | Oxidation (M) |
| Peptide Mass Tolerance | 10 |
| Peptide Mass Tolerance Units | ppm |
| Fragment Mass Tolerance | 0.5 |
| Fragment Mass Tolerance Units | Da |
| Mass values | Monoisotopic |
| Instrument type | Default |
| Isotope error mode | 0 |
| Decoy database also searched | 1 |
|  |  |
| **Format parameters** |  |
|  |  |
| Significance threshold | 0.00195 |
| Max. number of hits | 0 |
| Use MudPIT protein scoring | 1 |
| Ions score cut-off | 0.05 |
| Include same-set proteins | 1 |
| Include sub-set proteins | 1 |
| Include unassigned | 0 |
| Require bold red | 0 |
| Use homology threshold | 1 |
| Group protein families | 1 |
| Re-score using Percolator | 0 |
|  |  |
|  |  |
|  |  |
|  |  |
|  |  |
